# Genome-Wide Identification of the *TCP* Gene Family in Tetraploid Potato and Analysis of Their Potential Roles in Tuber Development

**DOI:** 10.3390/ijms27208995

**Published:** 2026-10-10

**Authors:** Yaping Li, Longbin Mei, Yongzhen Ma, Long Zhao, Jian Wang, Fang Wang

**Affiliations:** 1 Academy of Agriculture and Forestry Sciences, Qinghai University, Xining 810016, China; 15288463989@163.com (Y.L.); zhaolong0322@yeah.net (L.Z.); jianwang2197@163.com (J.W.); 2 Laboratory for Research and Utilization of Qinghai-Tibet Plateau Germplasm Resources, Qinghai University, Xining 810016, China; 3 Key Laboratory of Qinghai-Tibet Plateau Biotechnology, Ministry of Education, Qinghai University, Xining 810016, China; 4 Qinghai Provincial Key Laboratory of Potato Breeding, Qinghai University, Xining 810016, China; 5 College of Agriculture and Animal Husbandry, Qinghai University, Xining 810016, China

**Keywords:** gene expression patterns, genome-wide identification, phylogenetic analysis, TCP transcription factors, tuber development

## Abstract

Potato tuber formation is a complex developmental process regulated by coordinated interactions among multiple phytohormones and genes. TEOSINTE BRANCHED1/CYCLOIDEA/PROLIFERATING CELL FACTOR (TCP) transcription factors play important roles in plant growth, development and responses to environmental stimuli; however, their specific functions in tuber development in tetraploid potato remain poorly understood. In this study, we performed genome-wide identification and systematic characterization of the *TCP* gene family in tetraploid potato, integrated transcriptome profiling, quantitative real-time PCR (qRT-PCR), protein–protein interaction prediction and weighted gene co-expression network analysis (WGCNA) to identify candidate genes potentially associated with tuber development. A total of 57 *StTCP* genes were identified across 29 chromosomes and classified into eight subfamilies. Members within the same subfamily generally shared similar gene structures, conserved motifs and protein domains, whereas their promoter regions contained abundant *cis*-acting elements associated with light and phytohormone responses, growth and development, and stress responses. Expression profiling revealed distinct *StTCP* expression patterns under temperature stress and across tuber developmental stages. Eight *StTCP* genes exhibited pronounced stage-specific expression across the four developmental stages, and their expression trends were broadly supported by quantitative real-time PCR (qRT-PCR). In particular, *StTCP42*, *StTCP55*, and *StTCP57* showed significantly higher expression at the stolon hook stage than at the other stages (*p* < 0.05), whereas *StTCP20* was significantly upregulated at the subapical swelling stage (*p* < 0.05). Weighted gene co-expression network analysis (WGCNA) of RNA-seq data from the four developmental stages identified 27 co-expression modules. The turquoise, dark red, pink, and green modules were significantly and positively associated with the stolon hook, subapical swelling, initial tuber formation, and tuber formation stages, respectively; the turquoise module showed the strongest positive correlation with the stolon hook stage (r = 0.997, false discovery rate (FDR) = 2.77 × 10^−10^). Notably, *StTCP42* and *StTCP57* were both highly expressed at the stolon hook stage and were assigned to the turquoise module. *StTCP42* further exhibited high module membership (kME = 0.930) and gene significance (GS = 0.937, *p* = 6.79 × 10^−6^), providing convergent evidence for its association with early tuber development by the transcriptomic, qRT-PCR, and co-expression evidence. This study systematically characterised the *TCP* gene family in tetraploid potato and identified several candidate genes potentially associated with tuber development, particularly *StTCP42* and *StTCP57*. These findings provide a foundation for future functional studies aimed at elucidating the roles of TCP transcription factors in potato tuber formation and development.

## 1. Introduction

Potato (*Solanum tuberosum* L.) is an important food and economic crop whose yield depends largely on the initiation and development of underground tubers. Tubers are specialised storage organs derived from underground stolons and serve as major sinks for photosynthetic assimilates as well as organs for vegetative propagation [1]. Tuber development proceeds through several distinct stages, including the stolon hook stage, subapical swelling stage, initial tuber formation stage, and tuber formation stage, and involves coordinated physiological processes such as cell division, cell expansion, and nutrient accumulation [2,3]. Tuber formation is regulated by multiple endogenous and environmental factors, including photoperiod [4], temperature [5], nutrient availability [6,7] and phytohormones [8,9,10,11]. Recent studies have identified several key regulatory components involved in this process. For example, SELF-PRUNING 6A (*StSP6A*), a mobile signalling protein produced in leaves and transported to stolons, promotes tuber formation. In addition, the mobile regulator *StBEL5* promotes tuberisation, in part by modulating *StSP6A* expression [12,13].

Transcription factors (TFs) are sequence-specific DNA-binding proteins that play central roles in the regulation of gene expression in eukaryotes. By binding to *cis*-acting elements in the promoter or enhancer regions of target genes, TFs can activate or repress RNA polymerase II-mediated transcription and thereby modulate gene expression [14]. In plants, TFs also act as key regulatory nodes that integrate endogenous and environmental signals to coordinate growth, development, and stress responses [15]. Several major TF families, including TCP [16], MYB (v-Myb avian myeloblastosis viral oncogene homolog) [17] and NAC (NAM, ATAF1/2, and CUC2) [18] families, participate in diverse biological processes, including organ morphogenesis, phytohormone signalling, and adaptation to abiotic stresses such as drought, salinity, alkalinity and extreme temperatures.

The TCP (TEOSINTE BRANCHED1/CYCLOIDEA/PROLIFERATING CELL FACTOR) family comprises plant-specific transcription factors [19]. Based on structural features and phylogenetic relationships, TCP proteins are broadly classified into two major classes: Class I (PCF) and Class II, with the latter further divided into the CIN and CYC/TB1 subgroups. The primary structural difference between the two classes is that the TCP domain of Class I proteins lacks four amino acid residues that are present in Class II proteins. In addition, Class I TCP proteins generally contain a single conserved domain [20].

Accumulating evidence indicates that TCP transcription factors participate in diverse developmental processes in plants, including seed germination [21], floral and lateral branch development [22], secondary metabolite biosynthesis [23] and anthocyanin regulation [24].

Compared with model species, research on the TCP transcription factor family in potato remains relatively limited. Early studies showed that *StTCP1* exhibits a distinct expression pattern during axillary bud dormancy and is involved in the regulation of apical dominance, suggesting a potential role in the control of vegetative growth in potato [25]. With the increasing availability of potato genomic resources, systematic analyses of the *StTCP* gene family have been conducted. For example, Bao et al. identified 23 *TCP* genes in potato through genome-wide analysis and investigated their potential functions based on phylogenetic relationships, gene structures and expression patterns. Subsequent studies have shown that *StTCP* family members are involved not only in potato growth and development but also in plant immune responses. Notably, *StTCP23* has been implicated in the regulation of potato growth and defence responses to pathogens [26]. Nevertheless, the evolutionary characteristics, structural diversity, regulatory patterns and potential functions of the *TCP* gene family during tuber development remain incompletely understood. In particular, given the complex genomic background of tetraploid potato, a more comprehensive characterisation of *TCP* family members and their associations with tuber development is therefore warranted.

In recent years, the availability of high-quality tetraploid potato reference genomes has provided valuable resources for the systematic investigation of complex gene families. Building on these genomic resources, we performed a genome-wide identification of the TCP transcription factor family in tetraploid potato and characterised the chromosomal distribution, gene structures, conserved domains, phylogenetic relationships, gene duplication events and promoter *cis*-acting elements of the identified *StTCP* genes. In addition, expression profiles across different tissues and tuber developmental stages were integrated to investigate the potential roles of *StTCP* genes in potato growth, development and tuber formation. This study aimed to provide a comprehensive understanding of the *TCP* gene family in tetraploid potato and lays the foundation for further functional investigation of TCP transcription factors associated with tuber development.

## 2. Results

### 2.1. Identification of Gene Family Members and Protein Characteristics

**Figure 1:**
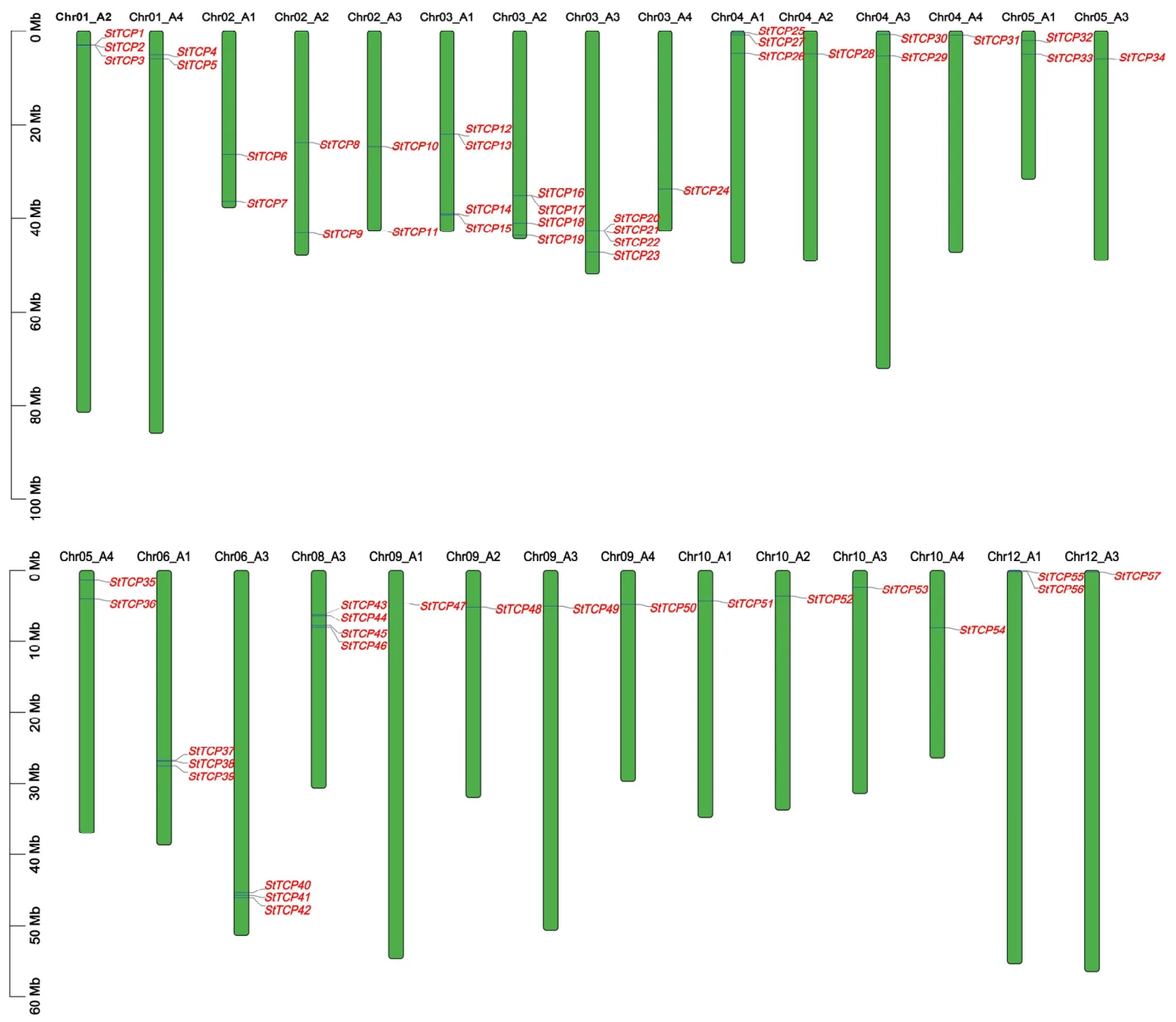
Chromosomal distribution of *StTCP* gene family members. Green bars represent the chromosomes of tetraploid potato, with chromosome numbers and chromosome lengths (Mb) indicated.

The predicted Q9 proteome was searched against the TCP-domain HMM profile (PF03634) using HMMER, resulting in the identification of 57 putative *StTCP* genes. These genes were distributed across 29 chromosomes, corresponding to 10 of the 12 chromosomes in the haploid chromosome set (2n = 4x =48; x = 12; Figure 1). After merging the homeologous chromosomes on the basis of the haploid chromosome set, Chr03 contained the largest number of *StTCP* genes (14, 24.6%), whereas no members were identified on Chr07 or Chr11; the other nine chromosomes each contained 3–7 genes, and both gene number and chromosomal position varied among chromosomes. The 57 genes were designated *StTCP1*–*StTCP57* according to their chromosomal position.

Physicochemical characterization of the corresponding StTCP proteins using ExPASy (Expert Protein Analysis System) revealed considerable variation in protein length, molecular weight and theoretical isoelectric point (Supplementary Table S1). The StTCP proteins ranged from 86 to 611 amino acids in length, with corresponding coding sequence (CDS) lengths ranging from 261 to 1836 bp. Their predicted molecular weights ranged from 9.92 to 64.69 kDa, whereas their theoretical isoelectric points (pI) ranged from 5.11 to 10.74. In total, 86% of the StTCP proteins had instability index values > 40. In addition, all StTCP proteins exhibited negative GRAVY (Grand average of hydropathicity) values, and 71% of the proteins had theoretical pI values > 7.

### 2.2. Construction of the Phylogenetic Tree and Subfamily Classification of the StTCP Gene Family

To investigate the evolutionary relationships of StTCP proteins and their phylogenetic affinities with TCP proteins from other plant species, a phylogenetic analysis was conducted using 57 StTCP proteins together with TCP proteins from four previously reported species. The dataset comprised 185 TCP proteins in total, including 24 AtTCP, 46 ZmTCP, 36 SlTCP, and 22 OsTCP proteins; the corresponding protein sequences are provided in Supplementary Table S2.

**Figure 2:**
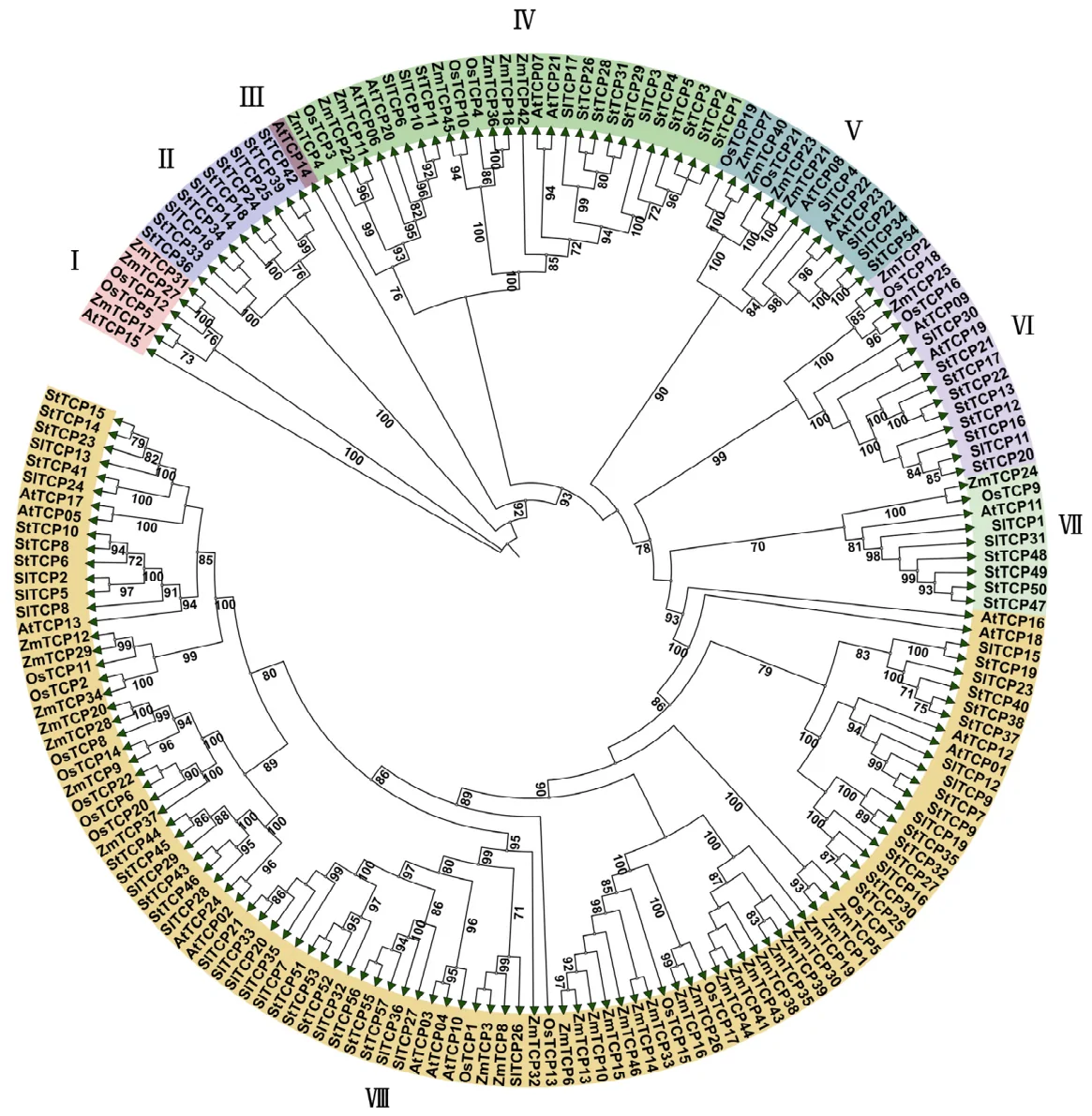
Phylogenetic tree of the *TCP* gene family across five plant species. SH-like support values calculated by FastTree are shown at the internal nodes of the tree; values ≥ 70 indicate reliable phylogenetic relationships. The eight *TCP* subfamilies are designated by Roman numerals I–VIII and distinguished by different colors. St, *Solanum tuberosum*; Zm, *Zea mays*; At, *Arabidopsis thaliana*; Os, *Oryza sativa*; Sl, *Solanum lycopersicum*.

A maximum-likelihood (ML) phylogenetic tree was constructed using FastTree 2.1.11 (Figure 2). SH-like (Shimodaira–Hasegawa-like) support values calculated by FastTree were used to evaluate internal branch support. Most internal branches showed relatively high support, supporting the overall stability of the inferred topology.

Based on the phylogenetic topology and the established classification of the *Arabidopsis* TCP family, the 185 TCP proteins were classified into eight subfamilies (I–VIII). The distribution of TCP proteins among these subfamilies differed among species. In potato, StTCP proteins were predominantly distributed in subfamilies II, IV, VI, VII and VIII. Subfamily VIII contained the largest number of StTCP proteins, comprising 28 members and accounting for the largest proportion of the StTCP family.

### 2.3. Gene Structure, Protein Structure and Conserved Motifs

**Figure 3:**
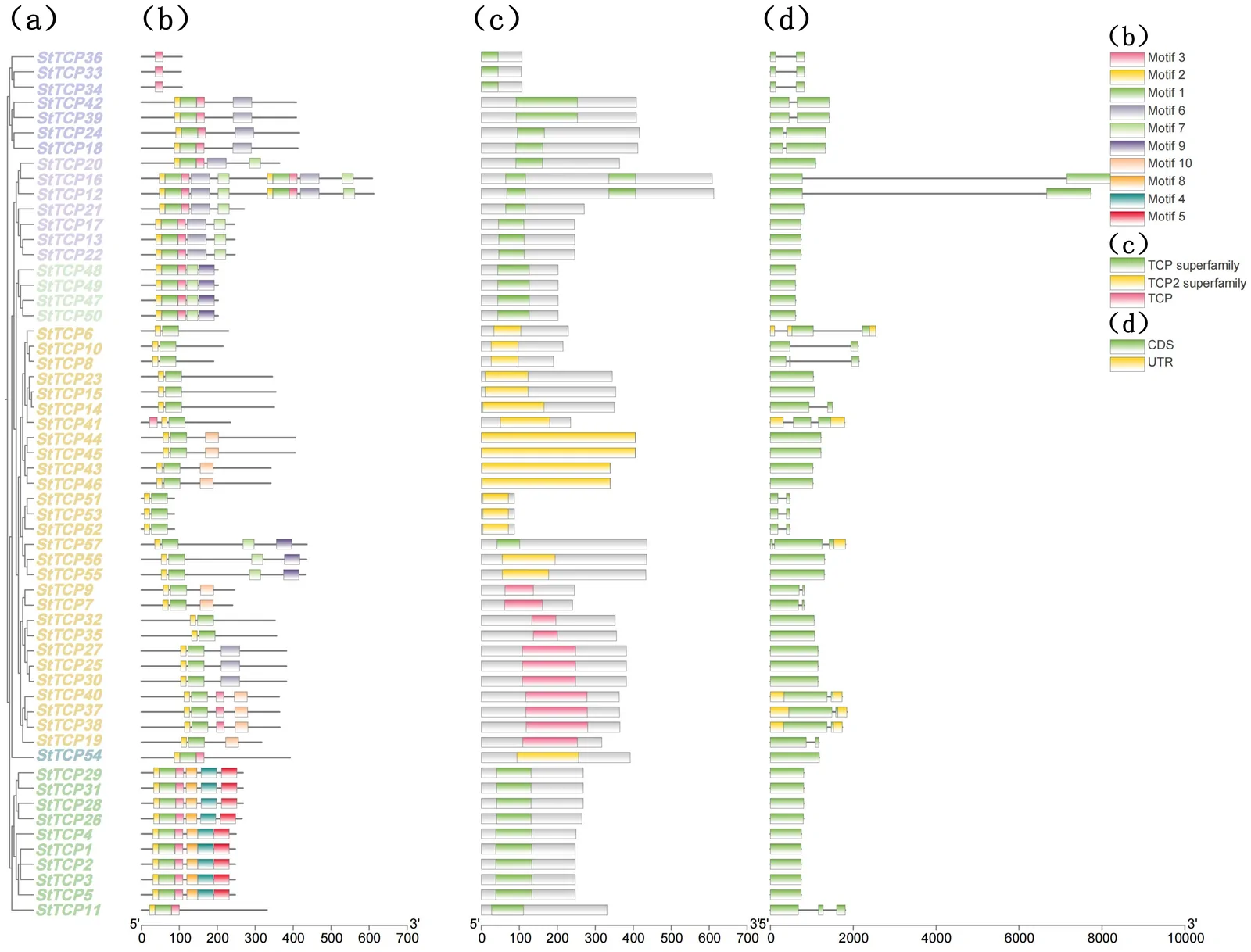
Phylogenetic tree, conserved motifs, protein domains and gene structure analysis of *StTCP* family members. (**a**) Phylogenetic tree of the 57 *StTCP* family members, Different colors in panel (**a**) indicate different phylogenetic subfamilies. (**b**) Conserved motifs of StTCP proteins, indicated by different colors, with the corresponding motif information shown in the legend in the upper-right corner. (**c**) Conserved protein domains of StTCP proteins. (**d**) Gene structure analysis of *StTCP* family members. Green rectangles, yellow rectangles and gray lines represent exons, untranslated regions (UTRs) and introns, respectively.

To characterise the structural features of the *StTCP* gene family, exon–intron organisation, conserved protein domains, and conserved motifs were systematically analysed. As shown in Figure 3d, 32 *StTCP* genes consisted of a single exon, 22 contained two exons, and three contained three exons. Genes within the same phylogenetic subfamily generally displayed similar exon–intron structures. For example, in subfamily IV, all members except *StTCP11* contained only one exon, whereas all members of subfamily II contained two exons.

Conserved domain analysis showed that 29 StTCP proteins contained a TCP superfamily domain, 17 contained a TCP2 superfamily domain and 11 contained a TCP domain; no member was found to contain two or more of these domains simultaneously (Figure 3c). Conserved motif analysis of the StTCP proteins using MEME (Multiple EM for Motif Elicitation) identified a total of 10 conserved motifs (Supplementary Table S3). Of these, 54 members contained both motif 1 and motif 2, whereas *StTCP33*, *StTCP34* and *StTCP36* lacked these two predominant motifs and displayed distinct motif compositions (Figure 3b).

### 2.4. Cis-Acting Element Analysis

**Figure 4:**
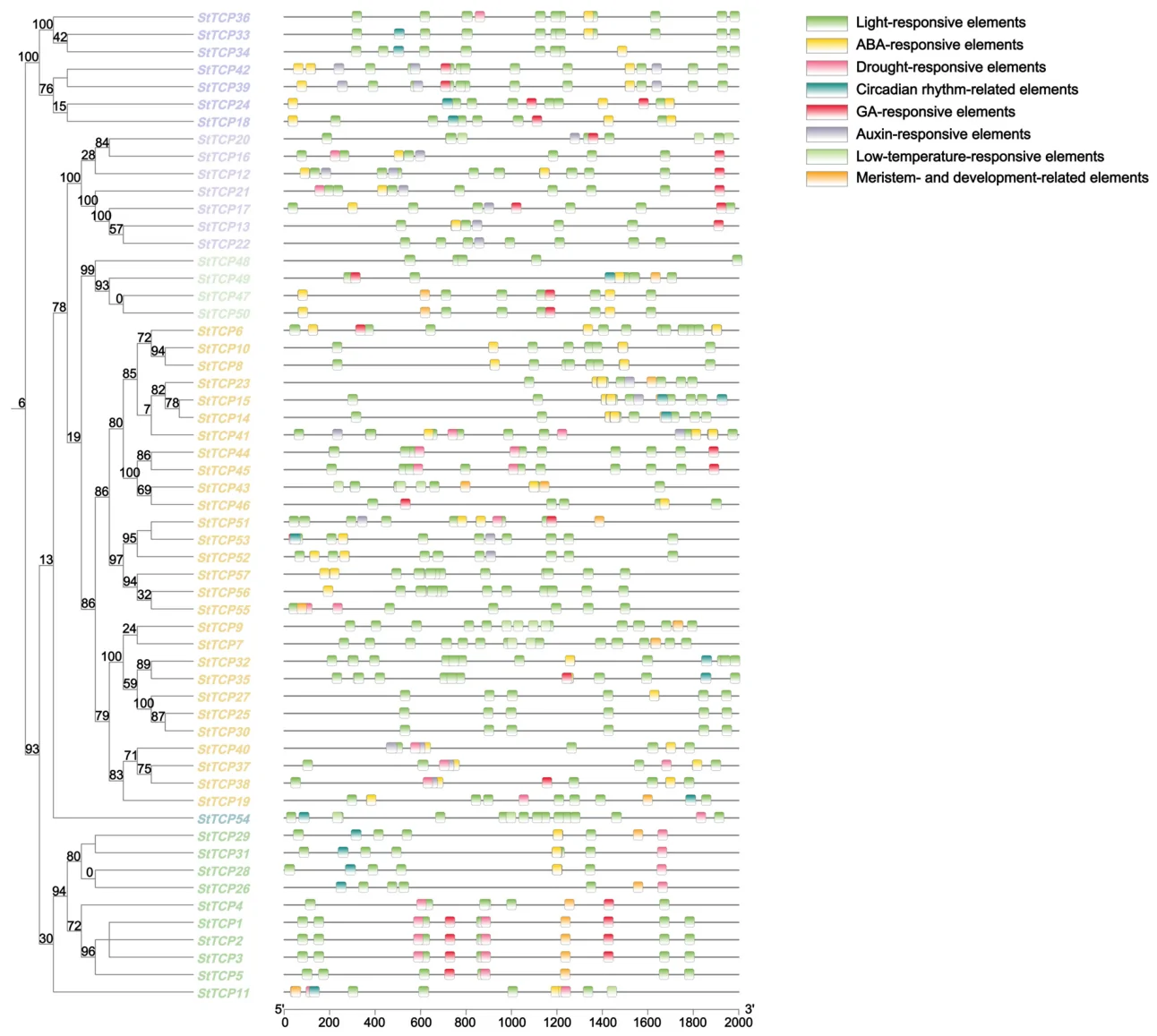
Prediction of *cis*-acting regulatory elements. Different colors represent different categories of *cis*-acting regulatory elements, including light-responsive elements, hormone-responsive elements, stress-responsive elements, circadian rhythm-related elements, and meristem- and development-related elements.

To investigate the potential transcriptional regulation of *StTCP* genes, *cis*-acting regulatory elements were predicted within the 2-kb promoter regions upstream of the transcription start sites of all 57 *StTCP* genes. The corresponding promoter sequences are provided in Supplementary Table S4. A total of 28 types of *cis*-acting elements were identified and classified by function into categories related to light response, phytohormone response, abiotic stress response, circadian rhythm, and meristem and development (Figure 4; Supplementary Table S5). Among them, light-responsive elements were the most abundant, comprising 17 types and occurring in the promoters of all 57 genes, mainly including Box 4, G-box, GT1-motif, GATA-motif, TCT-motif, and MRE. Among the phytohormone-responsive elements, the ABA-responsive element (ABRE) was present in the promoters of 40 genes (~70%), gibberellin-responsive elements (P-box, GARE-motif, TATC-box) in 26 genes, and auxin-responsive elements (TGA-element, etc.) in 18 genes. Among the abiotic stress-responsive elements, the drought-responsive MBS element was present in 23 genes, whereas the low-temperature-responsive LTR element was detected in six genes. In addition, circadian rhythm-related elements were identified in 17 genes, and meristem- and development-related elements (CAT-box; 19 genes) were also detected.

### 2.5. Gene Duplication Events and Synteny Analysis

**Figure 5:**
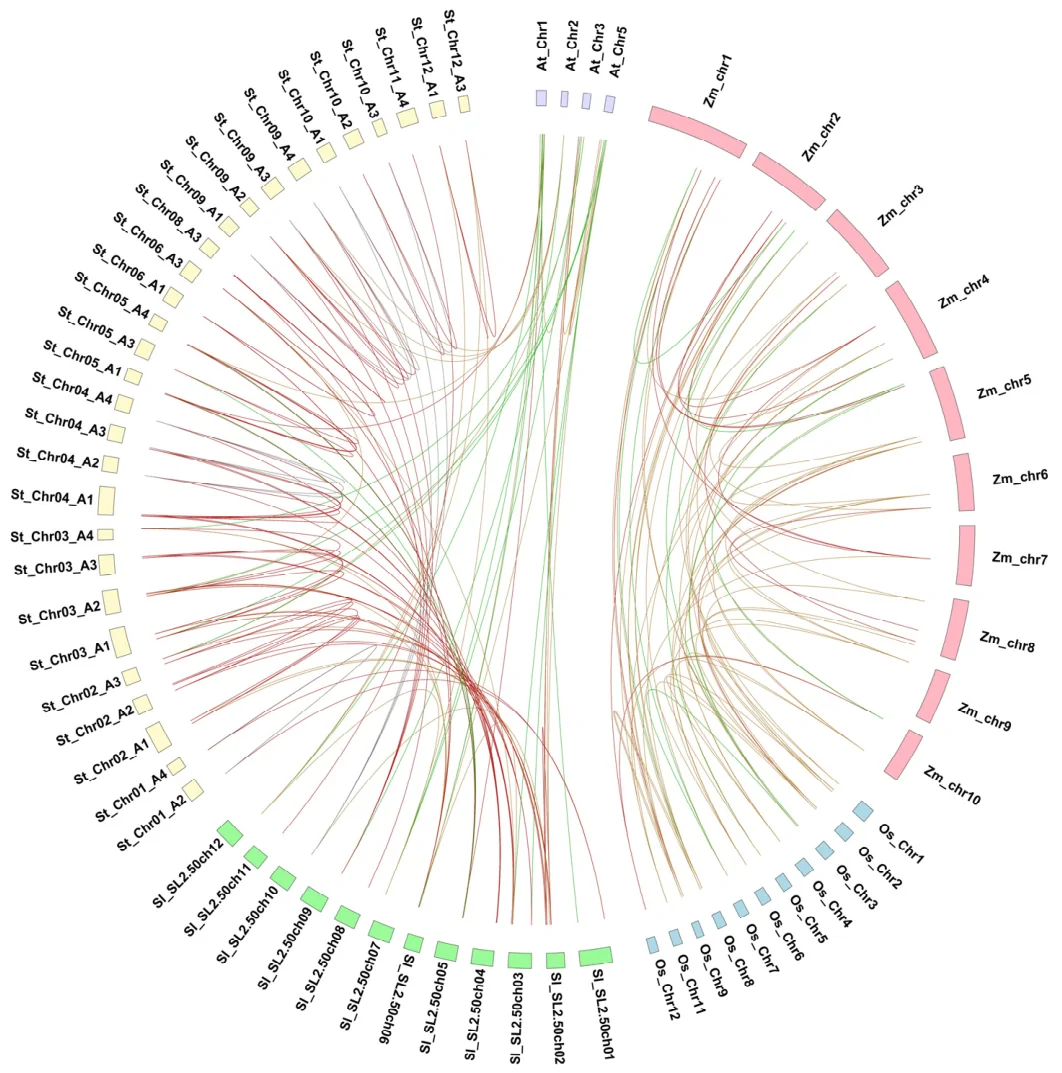
Intraspecific and interspecific collinearity. St: *Solanum tuberosum*; Zm: *Zea mays*; At: *Arabidopsis thaliana*; Os: *Oryza sativa*; Sl: *Solanum lycopersicum.* Green: ≤50% synteny between two gene pairs; orange: ≤75% synteny between two gene pairs; red: ≤99% synteny between two gene pairs.

To assess the evolutionary conservation and lineage-specific diversification of the *TCP* gene family, synteny analysis was performed among five representative plant species spanning eudicot and monocot lineages: *Arabidopsis thaliana* (At), *Solanum lycopersicum* (Sl) and *Solanum tuberosum* (St) were included as eudicot representatives, whereas *Oryza sativa* (Os) and *Zea mays* (Zm) represented monocots. Among these, tomato was included as a close relative of potato within the Solanaceae, whereas the remaining species represent more distantly related lineages, enabling comparison of *TCP* gene conservation and divergence across both closely and distantly related taxa. A total of 203 syntenic gene pairs were identified (Figure 5; Supplementary Table S6), of which 58.6% were interspecific and 41.4% were intraspecific.

The largest number of interspecific syntenic gene pairs was observed between tomato (Sl) and potato (St), with 53 pairs accounting for 44.5% of all interspecific syntenic relationships. Among the monocots, 41 syntenic gene pairs were identified between rice and maize. By contrast, no direct syntenic gene pairs were detected between the eudicot species (At, Sl, and St) and the monocot species (Os and Zm) under the criteria used in this analysis.

### 2.6. Expression Pattern Analysis of StTCP Genes

**Figure 6:**
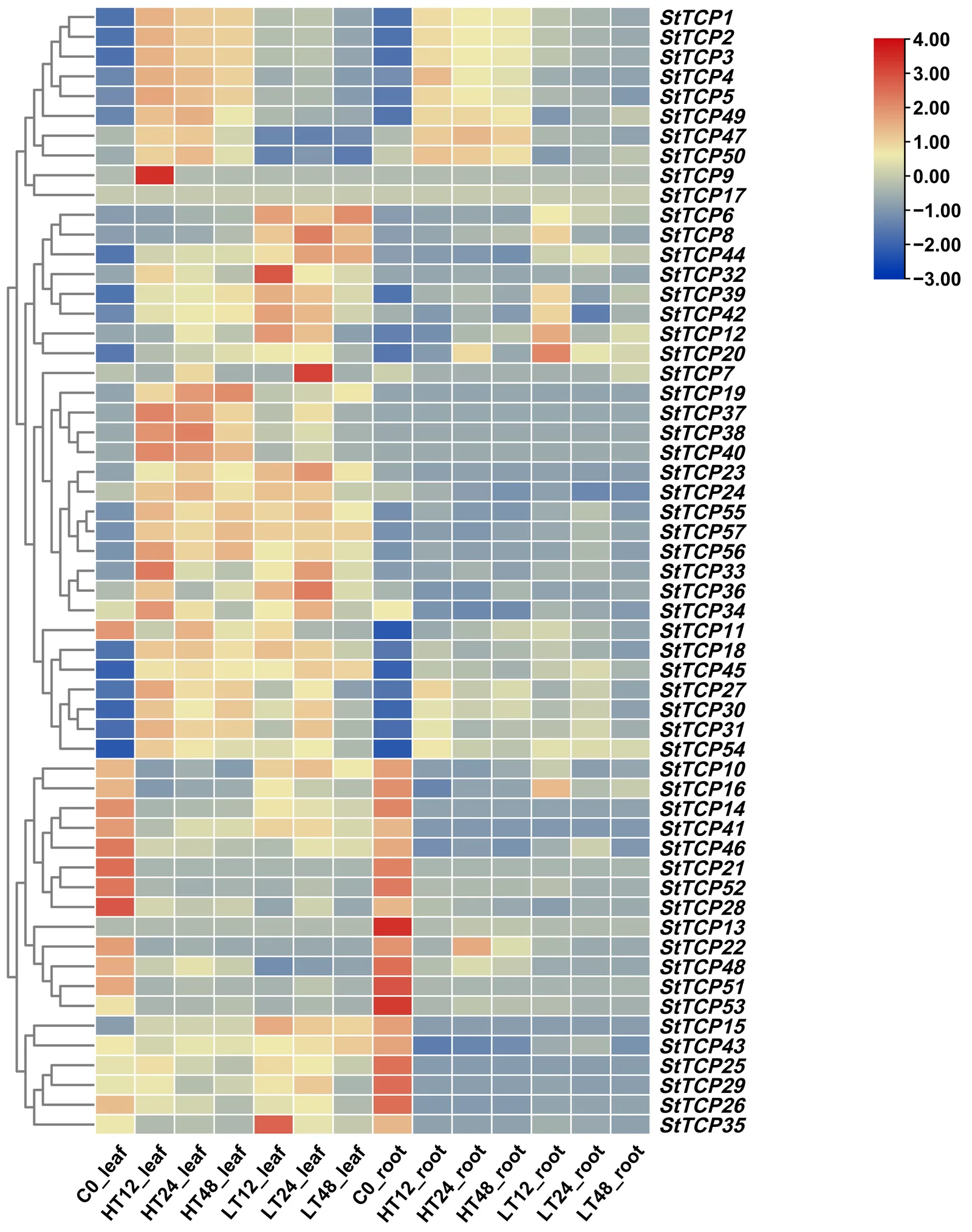
Expression patterns of *StTCP* gene family members in different organs under high-temperature (HT) and low-temperature (LT) stress treatments. HT, high-temperature treatment; LT, low-temperature treatment; 12, 24 and 48 h, treatment durations; Leaf, leaf tissue; Root, root tissue; C0, the corresponding untreated control samples collected before temperature treatment. Heatmap values represent FPKM (Fragments per kilobase of transcript per million mapped reads) expression values, log2-transformed.

Based on publicly available transcriptomic data from the National Genomics Data Center (NGDC), the expression profiles of *StTCP* genes in leaves and roots under high- and low-temperature treatments were analysed (Figure 6; Supplementary Table S7). Overall, *StTCP* genes displayed distinct tissue- and temperature-dependent expression patterns, with several genes showing lower expression under temperature treatments than in the corresponding controls. *StTCP19* and *StTCP38* were relatively highly expressed in leaves under both high- and low-temperature conditions. *StTCP1*–*StTCP5* generally showed higher expression under high-temperature treatment than under low-temperature treatment, with transcript abundance tending to decline with prolonged exposure to high temperature. Several genes exhibited particularly distinct patterns: *StTCP17* was not detectably expressed in any tissue or treatment, *StTCP21* was expressed predominantly in the control samples, and *StTCP9* was detected only in leaves after 12 h of high-temperature treatment.

**Figure 7:**
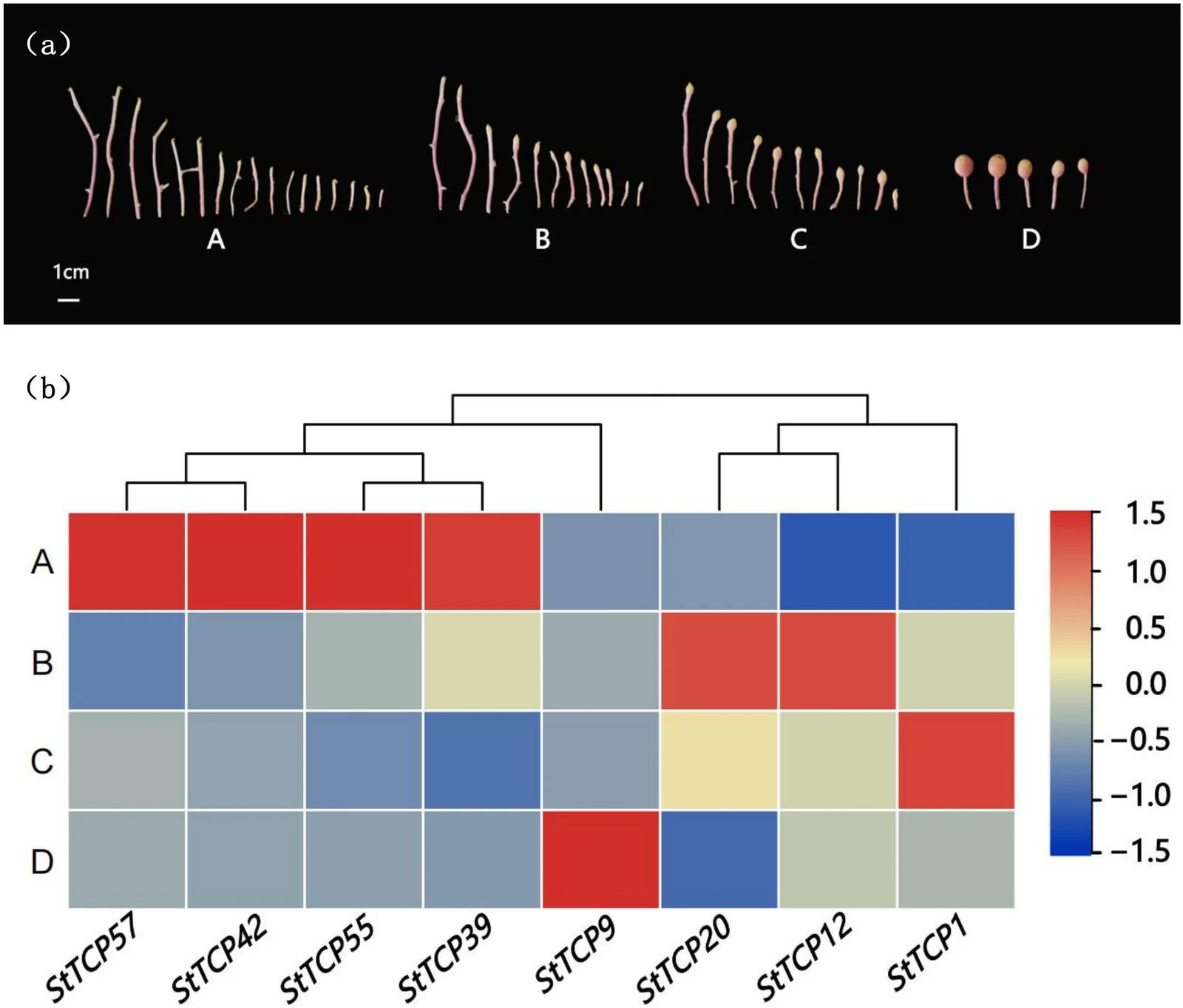
Morphological characteristics of potato tubers at four developmental stages and heatmap of the expression patterns of eight differentially expressed StTCP genes across these stages. (**a**) Morphological characteristics of potato tubers at different developmental stages. (**b**) Heatmap showing the expression patterns of eight differentially expressed *StTCP* genes across the four stages of tuber development. A: stolon hook stage; B: subapical swelling stage; C: initial tuber formation stage; D: tuber formation stage. In panel (**b**), the color scale represents relative expression levels, with red and blue indicating higher and lower expression levels, respectively.

The expression dynamics of *StTCP* genes during potato tuber development were further investigated. Based on morphological characteristics, tuber development was classified into four stages: the stolon hook stage, subapical swelling stage, initial tuber formation stage and tuber development stage (Figure 7a). Transcriptome analysis identified eight *StTCP* genes with distinct expression patterns across these developmental stages (Figure 7b; Supplementary Table S8) [27]. Among them, *StTCP39*, *StTCP42*, *StTCP55* and *StTCP57* showed their highest expression at the stolon hook stage and remained at relatively low levels during the subsequent stages. *StTCP12* and *StTCP20* reached peak expression at the subapical swelling stage, followed by a gradual decline during later development. *StTCP1* showed its highest expression level at the initial tuber formation stage, whereas *StTCP9* exhibited relatively high expression at the tuber development stage. Hierarchical clustering analysis further showed that *StTCP* genes with similar expression patterns were grouped into the same clusters.

### 2.7. qRT-PCR Validation of Eight Differentially Expressed StTCP Genes

**Figure 8:**
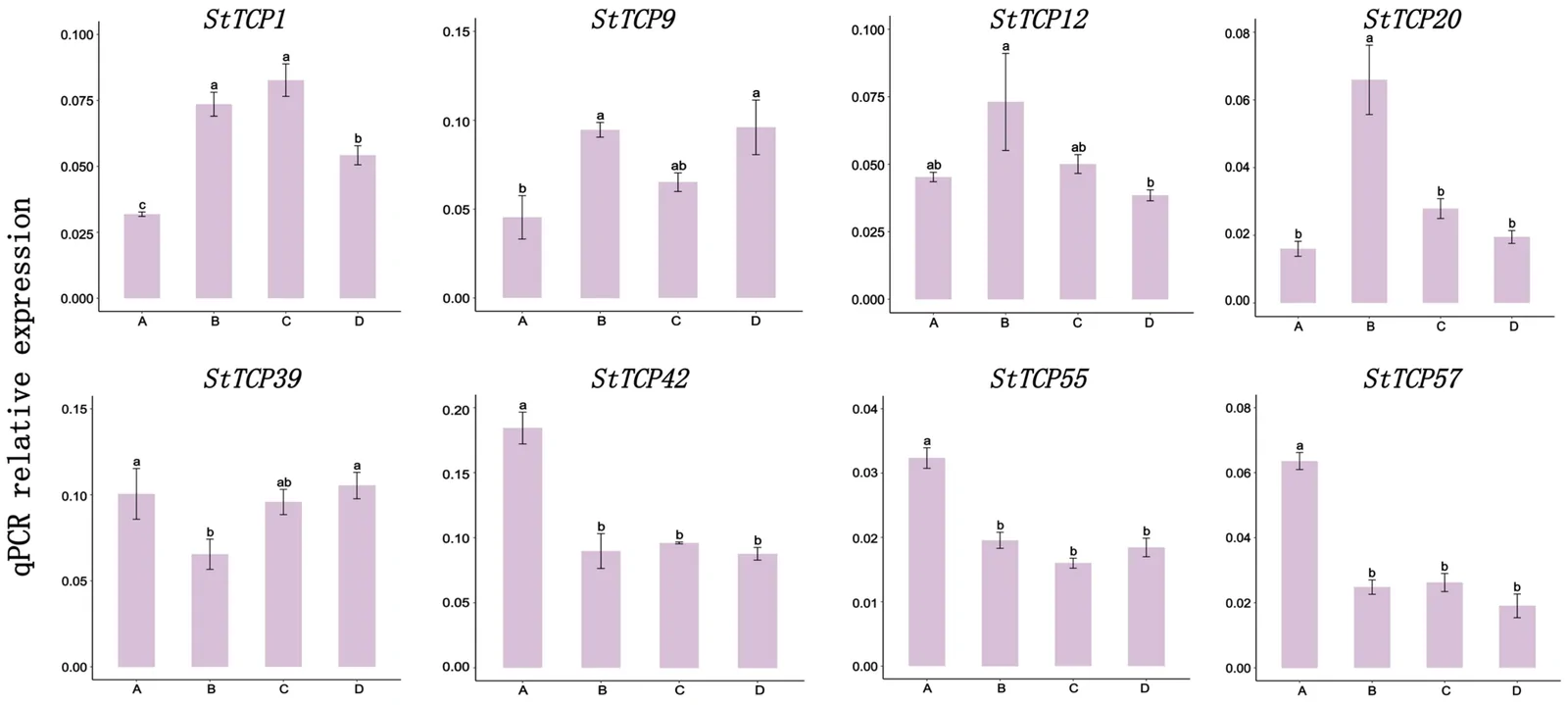
qRT-PCR validation of eight differentially expressed *StTCP* genes during potato tuber development. A, hook stage; B, subapical swelling stage; C, early tuber initiation stage; D, tuber initiation stage. Different lowercase letters indicate significant differences among treatments (*p* < 0.05).

To validate the transcriptomic expression profiles, eight *StTCP* genes exhibiting distinct stage-specific expression patterns during tuber development were selected for qRT-PCR analysis (Figure 8). Overall, the qRT-PCR results revealed distinct expression patterns among these genes, and were broadly consistent with the trends observed in the transcriptomic data. The expression levels of *StTCP42*, *StTCP55* and *StTCP57* at the stolon hook stage (A) were significantly higher than those at the other developmental stages (*p* < 0.05). *StTCP20* reached its highest expression level at the subapical swelling stage (B) and its expression was significantly higher than that at the other three stages (*p* < 0.05), while *StTCP12* also exhibited its highest expression level at this stage. *StTCP1* showed relatively high expression levels at both the subapical swelling stage and the initial tuber formation stage, whereas its expression was lowest at the stolon hook stage. *StTCP9* maintained relatively high expression levels at both the subapical swelling stage and the tuber formation stage. *StTCP39* exhibited its lowest expression level at the subapical swelling stage, while maintaining relatively high expression levels at the other developmental stages.

### 2.8. WGCNA Identification of StTCP Genes Associated with Tuber Developmental Stages

To further examine the associations between *StTCP* genes and tuber developmental stages at the co-expression network level, whole-transcriptome weighted gene co-expression network analysis (WGCNA) was performed using 12 RNA-seq samples representing four developmental stages: the stolon hook stage, subapical swelling stage, initial tuber formation stage and tuber formation stage. Sample clustering showed good consistency among the three biological replicates at each stage, with no obvious outlier detected (Supplementary Figure S1). Based on scale-free topology fit and network connectivity, a soft-thresholding power of β = 18 was selected to construct a signed co-expression network (Supplementary Figure S2). A total of 27 co-expression modules were identified, with module colors serving solely as arbitrary labels. The turquoise module contained the largest number of genes (Supplementary Figure S3; Supplementary Table S9). Module–trait relationships were assessed by correlating module eigengenes (MEs) with developmental stages, and modules with FDR < 0.05 were considered significantly stage-associated.

**Figure 9:**
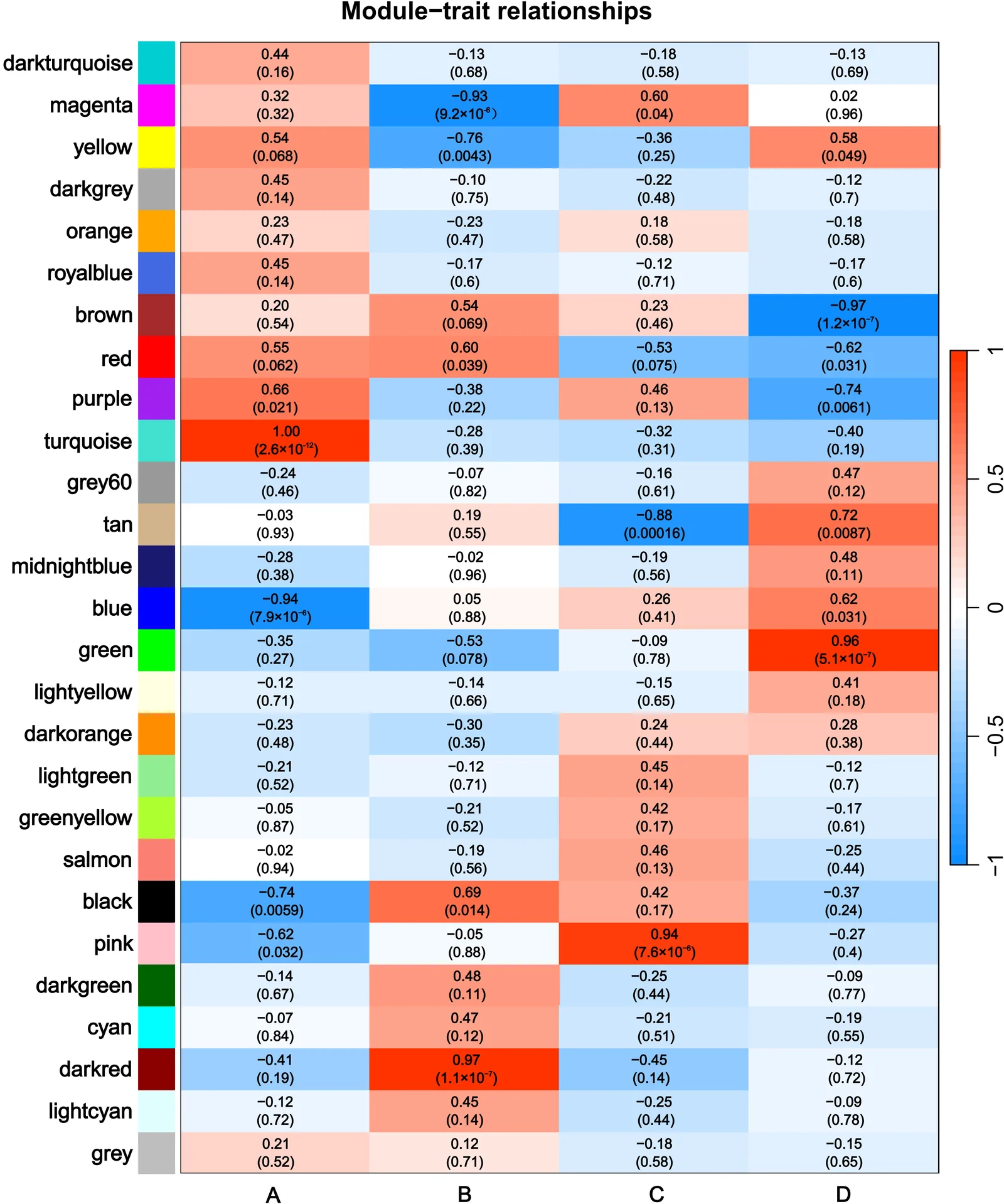
Heatmap of correlations between WGCNA modules and tuber developmental stages. A, stolon hook stage; B, subapical swelling stage; C, initial tuber formation stage; D, tuber formation stage.

Multiple modules showed significant associations with specific developmental stages (Figure 9). The turquoise module showed a strong positive correlation with the stolon hook stage (r = 0.997, FDR = 2.77 × 10^−10^), whereas the dark red module was positively correlated with the subapical swelling stage (r = 0.973, FDR = 4.29 × 10^−6^). The pink module showed a significant positive correlation with the initial tuber formation stage (r = 0.936, FDR = 1.42 × 10^−4^), while the green module was significantly positively associated with the tuber formation stage (r = 0.963, FDR = 1.37 × 10^−5^). In contrast, the blue, magenta, tan and brown modules showed significant negative correlations with the corresponding developmental stages. These results indicate that distinct co-expression modules are differentially associated with specific stages during potato tuber development.

Of the 57 identified *StTCP* genes, 33 met the expression-filtering criteria and were included in the WGCNA network analysis. These genes were distributed across multiple co-expression modules. The turquoise module contained *StTCP18*, *StTCP30*, *StTCP38*, *StTCP42*, and *StTCP57*; the pink module contained *StTCP5* and *StTCP54*; and the green module contained *StTCP46*. No *StTCP* genes that passed the expression filter were assigned to the dark red module (Table 1).

**Table 1:** *StTCP* Members in Modules Significantly Positively Correlated with Tuber Developmental Stages and Their Network Association Parameters.

TCP_Name	Module	Target_Stage	Module_ Stage_r	Module_ Stage_FDR	Own_kME	Target_ GS	Target_P
StTCP42	turquoise	stolon hook stage	0.997	0.000	0.930	0.937	0.000
StTCP18	turquoise	stolon hook stage	0.997	0.000	0.845	0.841	0.001
StTCP38	turquoise	stolon hook stage	0.997	0.000	0.834	0.796	0.002
StTCP30	turquoise	stolon hook stage	0.997	0.000	0.755	0.749	0.005
StTCP57	turquoise	stolon hook stage	0.997	0.000	0.625	0.629	0.028
StTCP54	pink	initial tuber formation stage	0.936	0.000	0.578	0.561	0.058
StTCP5	pink	initial tuber formation stage	0.936	0.000	0.495	0.487	0.109
StTCP46	green	tuber formation stage	0.963	0.000	0.758	0.684	0.014

**Figure 10:**
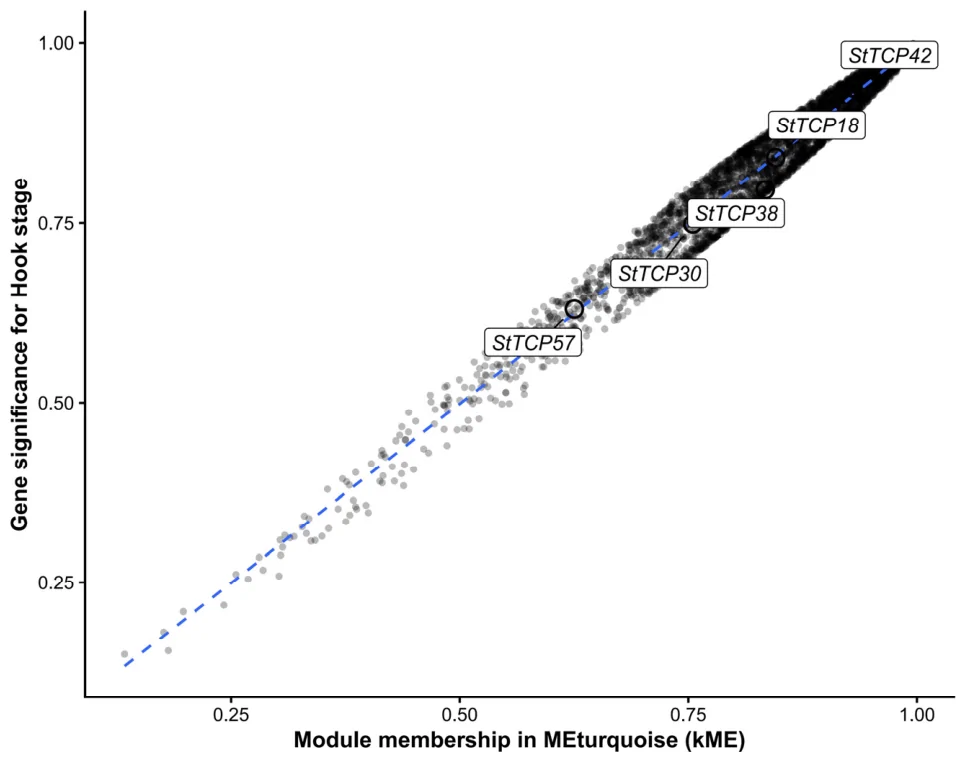
Scatter plot of module membership (kME) versus gene significance for the stolon hook stage in the MEturquoise module.

Because the turquoise module showed the strongest positive correlation with the stolon hook stage, module membership (kME; correlation with the module eigengene) and gene significance (GS) for this stage were further examined for the *StTCP* genes assigned to this module. Across the turquoise module, kME and GS were strongly correlated (r = 0.979). Among the *StTCP* genes in this module, *StTCP42* showed the highest module membership (kME = 0.930) and a high GS for the stolon hook stage (GS = 0.937, *p* = 6.79 × 10^−6^). *StTCP18* and *StTCP38* also exhibited relatively high module membership, with kME values of 0.845 and 0.834 and corresponding GS values of 0.841 and 0.796, respectively. *StTCP30* had kME and GS values of 0.755 and 0.749, respectively, whereas *StTCP57* had corresponding values of 0.625 and 0.629, with its association with the stolon hook stage reaching statistical significance (*p* = 0.028) (Figure 10).

Notably, the WGCNA results were partially concordant with the RNA-seq and qRT-PCR expression profiles described above. Both RNA-seq and qRT-PCR showed relatively high expression of *StTCP42* and *StTCP57* at the stolon hook stage, and both genes were assigned to the turquoise module, which was strongly and positively correlated with this stage. Among them, *StTCP42* exhibited high module membership and gene significance, providing particularly consistent support for its association with the stolon hook stage at both the expression and co-expression network levels. *StTCP57* also showed concordant evidence across the three analyses. Collectively, these results support *StTCP42* and *StTCP57* as candidate genes associated with the stolon hook stage, with the evidence being strongest for *StTCP42*. In addition, WGCNA highlighted *StTCP18* and *StTCP38* as further candidates associated with this developmental stage.

## 3. Discussion

Genome-wide identification and characterisation of gene families provides a valuable framework for investigating gene evolution and functional diversification and can facilitate the identification of candidate genes underlying agronomically important traits and their potential application in molecular breeding [28]. TCP transcription factors participate in diverse aspects of plant growth and development [29] and have been systematically characterized in numerous plant species, including *Arabidopsis thaliana* [30], *Oryza sativa* [31] and *Glycine max* [32].

In the present study, 57 *StTCP* genes were identified in the tetraploid potato genome using an HMMER-based search against the conserved TCP domain. This number is greater than those reported in previous studies of potato and several other Solanaceae species. Earlier studies identified 31 [33] and 23 [26] TCP genes in potato, while fewer members have been reported in tomato [34] and pepper [35]. These differences may arise from variation in reference genome assemblies and annotations, gene-identification criteria, and the retention of homeologous gene copies in the tetraploid genome. Chromosomal mapping further revealed an uneven distribution of the 57 StTCP genes, which may reflect the combined effects of gene duplication, chromosomal restructuring, and differential gene retention [36]. Thus, the relatively large number of TCP genes identified in this study may partly reflect the genomic complexity of tetraploid potato and the retention or expansion of TCP family members during genome evolution.

StTCP proteins exhibited considerable variation in physicochemical properties, including protein length, molecular weight and theoretical isoelectric point, indicating substantial diversity among family members. Most StTCP proteins showed relatively high instability indices and an overall hydrophilic nature, characteristics that may be consistent with the dynamic regulatory functions of transcription factors [37]. Nevertheless, the identified StTCP proteins retained TCP-related conserved domains, suggesting that the family remains largely conserved at the structural level despite variation in physicochemical properties. Several StTCP proteins differed markedly from other members in protein length and other physicochemical characteristics, which may reflect structural divergence during the evolutionary history of the *TCP* family. However, protein length, theoretical isoelectric point, instability index, and hydropathicity alone are insufficient to infer biological function, or functional loss. Therefore, these physicochemical differences should primarily be regarded as indicators of diversity within the *StTCP* family, and their functional significance requires further investigation in combination with conserved domain composition, expression patterns and experimental validation.

Phylogenetic analysis showed that potato TCP proteins clustered with their homologues from *Arabidopsis thaliana*, tomato, rice, and maize into several conserved clades, supporting substantial evolutionary conservation of the TCP family across plant lineages [38]. Nevertheless, the number of *StTCP* members differed markedly among subfamilies, suggesting that lineage-specific retention, expansion, and divergence may have occurred alongside the conservation of core sequence and structural features. Gene duplication and polyploidisation are widely recognised as important forces shaping the evolution of plant transcription factor families. Following duplication, gene copies may diverge in expression or regulatory properties and may subsequently undergo subfunctionalisation or neofunctionalisation. Thus, the relatively large number of members in certain TCP clades in tetraploid potato may partly reflect genome duplication and the retention of homeologous gene copies, potentially providing a genetic basis for subsequent functional diversification within the family [39].

Gene structure and conserved motif analyses further supported the structural conservation of the *StTCP* family. Most *StTCP* genes contained one or two exons, resulting in relatively compact gene structures, consistent with the low intron numbers commonly reported for *TCP* gene families in other plant species [40]. Members within the same phylogenetic subfamily generally exhibited similar exon–intron structures and motif compositions, indicating a relatively high degree of structural conservation. Most StTCP proteins contained the predominant motifs 1 and 2, suggesting that these motifs represent conserved sequence features of the family. However, several members displayed distinct structural characteristics. In particular, *StTCP33*, *StTCP34*, and *StTCP36* lacked motifs 1 and 2 and exhibited distinct motif compositions. These differences may reflect evolutionary divergence among family members, although their functional significance cannot be determined from structural features alone and requires further experimental investigation.

Analysis of promoter *cis*-acting elements further revealed the potential regulatory complexity of *StTCP* genes. The promoters of many family members contained multiple elements associated with light, phytohormone, and stress responses, suggesting that the *TCP* family may be coordinately regulated by developmental signals and environmental factors. Given that photoperiod and phytohormones are important regulators of potato tuber induction and development, the presence of these *cis*-acting elements suggests that *StTCP* genes may participate in tuber formation. Differences in *cis*-acting element composition among family members may also contribute to their tissue-specific and stage-specific expression patterns. However, *cis*-acting element prediction only indicates potential regulatory relationships and does not demonstrate direct interactions between specific transcription factors and corresponding regulatory elements. Further validation using promoter activity assays, yeast one-hybrid assays, chromatin immunoprecipitation followed by quantitative PCR (ChIP-qPCR), or related approaches will therefore be required.

Synteny analysis further supported both evolutionary conservation and lineage-specific diversification of the *StTCP* family. Conserved syntenic relationships among *TCP* genes were detected across multiple species, including *A. thaliana*, rice, tomato, potato and maize, consistent with substantial conservation of the family across divergent plant lineages. Gene duplication is an important driver of gene family evolution in plants, and duplicated genes may subsequently diverge in expression or regulatory properties through subfunctionalisation, neofunctionalisation, or other evolutionary processes, thereby contributing to functional and regulatory diversification [41].

Among the species examined in this study, *TCP* genes in tomato and potato showed a high degree of syntenic conservation, consistent with their close phylogenetic relationship within the Solanaceae. Previous studies have shown that Solanaceae species have retained numerous ancestral chromosomal blocks and relatively conserved genomic structures during evolution, contributing to high levels of synteny among closely related species [42]. Accordingly, the large number of syntenic relationships observed between tomato and potato *TCP* genes may reflect the long-term conservation of the *TCP* family within the Solanaceae. In contrast, no direct *TCP* syntenic gene pairs were detected between the eudicot and monocot species, which may reflect chromosomal rearrangements, gene loss and changes in genome structure following the divergence of these two major plant lineages [43].

Intragenomic syntenic relationships in potato further suggest that gene duplication and polyploidisation may have contributed to the relatively large number of *StTCP* family members. The autotetraploid potato genome contains multiple highly homologous chromosome sets, and the retention of homeologous gene copies may provide an important genomic basis for the maintenance and expansion of this gene family. Duplicated genes retained following polyploidisation may diverge in expression and regulatory properties through gene effects, subfunctionalisation, or neofunctionalisation [44], thereby increasing the potential regulatory diversity of gene families involved in complex developmental processes. Consequently, the retention of duplicated *StTCP* genes may have contributed not only to the size of the family but also to subsequent divergence in gene expression and function.

In addition to evolutionary and structural characteristics, expression profiles provide further clues regarding the potential functions of *TCP* family members. Transcriptomic analysis under high- and low-temperature treatments revealed distinct tissue- and temperature-dependent expression patterns among *StTCP* genes, suggesting that different family members may be differentially responsive to temperature conditions. More notably, several *StTCP* genes exhibited pronounced stage-dependent expression during tuber development. *StTCP39*, *StTCP42*, *StTCP55* and *StTCP57* were highly expressed at the stolon hook stage, whereas *StTCP12* and *StTCP20* showed relatively high expression at the subapical swelling stage. *StTCP1* and *StTCP9* exhibited distinct expression patterns during later developmental stages. The qRT-PCR results broadly supported the stage-specific trends observed in the RNA-seq data, particularly the relatively high expression of *StTCP42*, *StTCP55* and *StTCP57* at the stolon hook stage. Previous studies have shown that TCP transcription factors participate in cell proliferation, organ formation and phytohormone signalling [38], processes closely associated with the transition from stolon to tuber and subsequent tuber enlargement [45]. Thus, the stage-dependent expression patterns observed here suggest that different *StTCP* members may be associated with distinct phases of tuber development. However, their specific regulatory roles cannot be inferred from expression patterns alone. For example, the relatively high expression of *StTCP42*, *StTCP55*, and *StTCP57* at the stolon hook stage supports their association with early tuber development, but whether they directly participate in tuber induction, cell-cycle regulation, or meristem activity remains to be determined experimentally.

To further investigate the associations between *StTCP* genes and tuber developmental stages at the co-expression network level, WGCNA was performed using whole-transcriptome expression data. Distinct co-expression modules were significantly associated with the four developmental stages. Specifically, the turquoise, dark red, pink, and green modules were positively correlated with the stolon hook stage, subapical swelling stage, initial tuber formation stage, and tuber formation stage, respectively. These stage-associated modules indicate substantial differences in co-expression relationships across tuber development and provide a network-level context for prioritising *StTCP* genes potentially associated with specific developmental stages.

Importantly, the WGCNA results were only partially concordant with the RNA-seq and qRT-PCR findings, reflecting the different information captured by these approaches. RNA-seq and qRT-PCR characterise the expression patterns of individual genes across developmental stages, whereas WGCNA identifies groups of genes with coordinated expression across samples and evaluates their associations with specific traits or developmental stages. Therefore, a gene showing pronounced stage-specific expression is not necessarily assigned to the module most strongly associated with that stage. In the present study, *StTCP42* and *StTCP57* showed relatively high expression at the stolon hook stage in both RNA-seq and qRT-PCR analyses and were also assigned to the turquoise module, which was strongly and positively correlated with this developmental stage. In particular, *StTCP42* exhibited both high module membership and high gene significance for the stolon hook stage, providing the most consistent evidence of association across the expression and co-expression analyses. Although the network-level association of *StTCP57* was weaker, its expression profile and WGCNA assignment were broadly concordant with an association with the stolon hook stage. Taken together, the integration of RNA-seq, qRT-PCR, and WGCNA results prioritises *StTCP42* as a candidate gene for subsequent functional investigation of early tuber development.

WGCNA further highlighted *StTCP18*, *StTCP38* and *StTCP30* as members of the turquoise module associated with the stolon hook stage, whereas *StTCP46* was assigned to the green module associated with the tuber formation stage. Among these genes, *StTCP18* and *StTCP38* exhibited relatively high module membership and gene significance for the stolon hook stage, although they were not among the eight genes initially selected on the basis of stage-dependent expression patterns. These results underscore the complementarity of candidate-gene prioritisation based on individual expression profiles and co-expression network analysis.

Overall, this study systematically characterised the *TCP* gene family in tetraploid potato by integrating phylogenetic, structural, promoter, synteny, expression, qRT-PCR, and co-expression analyses. The results revealed substantial structural conservation within the *StTCP* family together with pronounced member-specific divergence in expression profiles and co-expression network associations. Among the identified genes, *StTCP42* showed the most consistent association with early tuber development across RNA-seq, qRT-PCR, and WGCNA analyses, highlighting it as a priority candidate for further functional investigation. *StTCP57* was also supported by multiple lines of evidence, whereas WGCNA additionally highlighted *StTCP18*, *StTCP38*, and *StTCP46* as candidates potentially associated with specific stages of tuber development.

The evidence obtained in the present study is primarily based on bioinformatic predictions, expression analyses and co-expression relationships and is therefore insufficient to establish direct regulatory roles for these *TCP* members in tuber formation. Moreover, the WGCNA network was constructed using 12 RNA-seq samples, which may limit the robustness and generalisability of the inferred module–stage associations. These relationships should therefore be further evaluated using additional biological replicates and independent transcriptomic datasets. Future studies integrating gene editing, overexpression or gene silencing, subcellular localisation, downstream target identification and protein–protein interaction assays will be required to validate the functions of candidate genes such as *StTCP42*, *StTCP57*, *StTCP18* and *StTCP38* and further elucidate the molecular mechanisms by which TCP transcription factors may contribute to potato tuber formation and development.

## 4. Materials and Methods

### 4.1. Identification and Basic Characterization of the TCP Gene Family in Potato

The TCP domain identifier (PF03634) was retrieved from the Pfam database, and the corresponding hidden Markov model (HMM) profile was downloaded [46]. Predicted protein sequences of the autotetraploid potato (*Solanum tuberosum* L.) cultivar Qingshu 9 (Q9 genome; PRJCA006096) were obtained from the BIG Data Center [47]. The Q9 predicted proteome was screened against the TCP domain HMM profile using hmmscan in HMMER v3.3.2 with default parameters. Proteins containing the conserved TCP domain were identified as members of the potato TCP transcription factor family [48]. The identified *TCP* genes were named according to their chromosomal locations.

The physicochemical properties of the encoded proteins, including protein length, molecular weight, pI, instability index, and grand average of hydropathicity (GRAVY), were calculated using the ExPASy ProtParam tool (https://www.expasy.org (accessed on 15 June 2026)) [49]. The chromosomal distribution and physical positions of the identified *StTCP* genes were visualised using TBtools-II v2.485 [50].

### 4.2. Phylogenetic Tree Construction

Genome assemblies, protein sequences, and gene annotation files (GFF3, General Feature Format) of the five species used for the phylogenetic and subsequent synteny analyses were obtained from public databases as follows: *Arabidopsis thaliana* TAIR10 from The *Arabidopsis* Information Resource (TAIR) [51]; the *Oryza sativa* genome annotation (IRGSP-1.0/MSU7) from Ensembl Plants [52]; the *Solanum lycopersicum* genome annotation (SL2.50/ITAG2.4) from the Sol Genomics Network (SGN) [53]; the *Zea mays* genome annotation (B73 RefGen_v4/AGPv4) from Ensembl Plants [54]; and the genome assembly and annotation of the autotetraploid potato cultivar ‘Qingshu No. 9’ from the Genome Warehouse (GWH) of the National Genomics Data Center (NGDC; China National Center for Bioinformation) under BioProject PRJCA006096 [47]. Protein-coding sequences were extracted from the corresponding genome assemblies according to the GFF3 annotation files and translated into protein sequences.

TCP proteins from *A. thaliana* (24 AtTCPs), *Z. mays* (46 ZmTCPs), *S. lycopersicum* (36 SlTCPs), and *O. sativa* (22 OsTCPs), together with the 57 StTCP proteins identified in this study, were used for phylogenetic analysis. Multiple sequence alignment of the 185 TCP protein sequences was performed using MUSCLE v5.1 (Multiple Sequence Comparison by Log-Expectation) [55]. A maximum-likelihood (ML) phylogenetic tree was subsequently constructed using FastTree with default parameters [56]. Internal branch support was assessed using SH-like (Shimodaira–Hasegawa-like) support values calculated by FastTree. The resulting tree was visualised and annotated using the Interactive Tree of Life (iTOL), and internal branches with SH-like support values ≥ 0.70 were displayed [57]. TCP proteins were assigned to subfamilies based on the established classification of the *A. thaliana* TCP family.

### 4.3. Analysis of Conserved Motifs and Gene Structure of StTCP Proteins

Conserved motifs in the identified StTCP proteins were predicted using MEME v4.11.2 [58], with the maximum number of motifs set to 10 and all other parameters left at the default settings. Conserved protein domains were annotated by using the NCBI Conserved Domain Database (CDD) through the online CD-Search tool [59]. Gene structure information, including coding sequences (CDSs), untranslated regions (UTRs), and introns, was extracted from the potato genome annotation file [47]. The phylogenetic tree was constructed as described in Section 4.2. The Newick-format phylogenetic tree, MEME motif results, CDD domain annotations, and gene structure information were then imported into the Gene Structure View module of TBtools-II v2.485 (Toolbox for Biologists) to jointly visualise phylogenetic relationships, conserved motifs, protein domains, and gene structures [50].

### 4.4. Analysis of Cis-Acting Elements in the Promoter Region

Using TBtools-II v2.485, the 2-kb sequences upstream of the *StTCP* genes were extracted from the genome of the autotetraploid potato cultivar Qingshu No. 9 and defined as putative promoter regions [47,50]. These promoter sequences were submitted to the PlantCARE database to predict cis-acting regulatory elements [60]. The predicted elements were subsequently classified according to their annotated functions and visualised using TBtools-II v2.485 to determine their distribution within the 2-kb promoter regions of the *StTCP* genes [50].

### 4.5. Synteny Analysis

The genome assemblies and gene annotation (GFF3) files used for the synteny analysis were the same as those described in Section 4.2 (phylogenetic analysis). Genome-wide sequence comparisons were performed using BLASTN v2.5.0 with an E-value threshold of 1 × 10^10^ to identify homologous gene pairs [61]. Syntenic blocks were subsequently detected using MCScanX implemented in TBtools-II v2.485 [50,62] and the intraspecific and interspecific syntenic relationships among *TCP* gene family members were visualised using the circlize package in R [63,64].

### 4.6. Expression Analysis of TCP Gene Family Members

RNA-seq data were obtained from the National Genomics Data Center (NGDC) and the NCBI Sequence Read Archive (SRA). The dataset used for temperature-response analysis was derived from the autotetraploid potato cultivar Qingshu No. 9 (BioProject PRJCA006877; Supplementary Table S7) and comprised transcriptomic data from leaf and root tissues collected at 0, 12, 24, and 48 h under high-temperature (HT) and low-temperature (LT) treatments [47]. To investigate *StTCP* expression patterns during tuber development, RNA-seq data representing four developmental stages—the stolon hook stage, subapical swelling stage, initial tuber formation stage, and tuber formation stage—were obtained from the NCBI SRA under BioProject PRJNA1436775 [27].

After the raw RNA-seq data were downloaded, sequencing quality was assessed using FastQC v0.11.9, and low-quality reads and adapter sequences were removed using fastp v0.23.4. The resulting high-quality reads were aligned to the reference genome of the autotetraploid potato cultivar Qingshu No. 9 [47], and gene expression levels were quantified based on the corresponding genome annotation file. Expression levels of *StTCP* genes were represented as fragments per kilobase of transcript per million mapped reads (FPKM). Expression profiles of the 57 *StTCP* genes across tissues, temperature treatments, and tuber developmental stages were extracted for subsequent analysis. Heatmaps were generated using TBtools-II v2.485 [50] to visualise the expression patterns of *StTCP* genes across the analysed conditions.

### 4.7. qRT-PCR Validation of Differentially Expressed StTCP Genes

The potato cultivar Qingshu No. 9 was used as the experimental material and grown in pots in a greenhouse at the Institute of Biotechnology, Qinghai University. Based on morphological characteristics, tuber development was divided into four stages: the stolon hook stage, subapical swelling stage, initial tuber formation stage, and tuber formation stage. Samples were collected 55 days after sowing, when stolons representing all four developmental stages were simultaneously available. For each developmental stage, nine plants were sampled. Tissues from three plants were pooled to form one biological replicate, resulting in three independent biological replicates per developmental stage. Approximately 3 g of tissue was collected for each biological replicate. The collected samples were immediately frozen in liquid nitrogen and stored at −80 °C until RNA extraction.

Total RNA was extracted from samples at various developmental stages using the TaKaRa MiniBEST Plant RNA Extraction Kit (Takara Bio Engineering (Dalian) Co., Ltd., Dalian, China) following the manufacturer’s instructions. First-strand cDNA was synthesized with PrimeScript™ RT Master Mix (Perfect Real Time) (Takara Bio Engineering (Dalian) Co., Ltd.) for real-time quantitative PCR according to the manufacturer’s protocol. The cDNA concentration was measured using a NanoDrop One spectrophotometer (Thermo Fisher Scientific, Beijing, China), and the cDNA was diluted to approximately 200 ng/μL for downstream analysis.

Based on the transcriptomic expression profiles, eight *StTCP* genes exhibiting distinct stage-specific expression patterns were selected for quantitative real-time PCR (qRT-PCR) analysis. qRT-PCR was performed using TB Green^®^ Premix Ex Taq™ II (Takara, Osaka, Japan) on a Roche LightCycler^®^ 96 Real-Time PCR System (Roche, Beijing, China) according to the manufacturer’s instructions. The potato Actin gene (GenBank accession X83206) [65] was used as the internal reference because of its relatively stable expression and widespread use in potato gene expression studies. Three biological replicates and three technical replicates were included for each sample. Relative expression levels were calculated using the 2^−ΔΔCt^ method [66]. Gene-specific primers were designed using the NCBI Primer-BLAST online tool (https://www.ncbi.nlm.nih.gov/tools/primer-blast/ (accessed on 15 June 2026)) [67]. Differences in relative gene expression among developmental stages were evaluated using one-way analysis of variance (ANOVA), followed by Duncan’s multiple range test for multiple comparisons. Differences were considered statistically significant at *p* < 0.05. The results were visualised using R [64].

### 4.8. Weighted Gene Co-Expression Network Analysis (WGCNA)

To identify candidate *StTCP* genes associated with potato tuber development, weighted gene co-expression network analysis (WGCNA) was performed using RNA-seq count data from 12 samples representing four developmental stages: the stolon hook stage (Hook), subapical swelling stage (Swelling), initial tuber formation stage (Early_tuber), and tuber formation stage (Tuber). Lowly expressed genes were first filtered by retaining genes with read counts ≥ 5 in at least three samples. The filtered count data were then subjected to variance-stabilising transformation (VST) using DESeq2 [68]. Based on expression variance, the 20,000 most variable genes were selected, and *StTCP* genes that passed the expression filter were additionally retained. A total of 20,030 genes were used for network construction.

Sample clustering and soft-thresholding power selection were performed using the WGCNA package in R v4.4.2 [64,69]. A soft-thresholding power of β = 18 was selected to construct a signed co-expression network. Co-expression modules were identified using the blockwiseModules function with the following principal parameters: minModuleSize = 30, deepSplit = 2, mergeCutHeight = 0.25, and maxBlockSize = 5000. Pearson correlation coefficients were calculated between module eigengenes (MEs) and the four developmental stages, and the corresponding *p* values were adjusted using the Benjamini–Hochberg procedure. Modules with a false discovery rate (FDR) < 0.05 were considered significantly associated with specific developmental stages. Finally, module membership (kME) and gene significance (GS) were evaluated for *StTCP* genes to prioritise candidate genes associated with tuber development.

## 5. Conclusions

In this study, 57 *StTCP* genes were identified and systematically characterised at the genome-wide level in tetraploid potato. Phylogenetic, gene structure, conserved motif, and synteny analyses revealed substantial structural conservation within the *StTCP* family together with evidence of evolutionary diversification, potentially associated with gene duplication, polyploidisation, and the retention of homeologous gene copies. Promoter *cis*-acting element analysis further indicated that *StTCP* genes may respond to multiple regulatory signals, including light, phytohormones, and environmental stresses. Expression profiling revealed pronounced divergence among *StTCP* members under temperature treatments and across tuber developmental stages. Eight genes exhibited distinct stage-specific expression patterns, which were broadly supported by qRT-PCR analysis. WGCNA identified 27 co-expression modules, of which the turquoise, dark red, pink, and green modules were significantly and positively associated with the stolon hook, subapical swelling, initial tuber formation, and tuber formation stages, respectively. *StTCP42* and *StTCP57* showed relatively high expression at the stolon hook stage and were assigned to the turquoise module, with *StTCP42* receiving the most consistent support across RNA-seq, qRT-PCR, and WGCNA analyses. In addition, *StTCP18*, *StTCP38*, and *StTCP46* emerged as further candidates associated with specific developmental stages. Collectively, these findings provide a systematic framework for understanding the *TCP* gene family in tetraploid potato and identify priority candidates for future functional investigation of tuber development. Further experimental validation will be required to determine their specific biological roles and regulatory mechanisms.

## Data Availability

The datasets analyzed in this study are publicly available. RNA-seq data for the tetraploid potato cultivar ‘Qingshu 9’ under high- and low-temperature treatments were obtained from the National Genomics Data Center (NGDC) BioProject database under accession number PRJCA006877 (https://ngdc.cncb.ac.cn/bioproject/browse/PRJCA006877 (accessed on 17 July 2026)). Transcriptome data covering four stages of potato tuber development were retrieved from the NCBI BioProject database under accession number PRJNA1436775 (https://www.ncbi.nlm.nih.gov/bioproject/PRJNA1436775 (accessed on 17 July 2026)). The tetraploid potato reference genome and gene annotation files used in this study were downloaded from the Spud DB database using BioProject accession number PRJNA753086 (http://spuddb.uga.edu/ (accessed on 17 July 2026); https://www.ncbi.nlm.nih.gov/bioproject/PRJNA753086 (accessed on 17 July 2026)). All data supporting the findings of this study are available from the corresponding public repositories. All data supporting the conclusions of this article are provided within the article and its Supplementary Information.

## Supplementary Materials

The following supporting information can be downloaded at: https://www.mdpi.com/article/10.3390/ijms27208995/s1.

## Abbreviations

BLAST	Basic Local Alignment Search Tool
cDNA	Complementary DNA
CDD	Conserved Domain Database
CDS	Coding sequence
ChIP-qPCR	Chromatin immunoprecipitation quantitative PCR
ExPASy	Expert Protein Analysis System
FDR	False discovery rate
FPKM	Fragments per kilobase of transcript per million mapped reads
GFF	General Feature Format
GRAVY	Grand average of hydropathicity
GS	Gene significance
HMM	Hidden Markov model
HT	High temperature (treatment)
iTOL	Interactive Tree Of Life
kDa	Kilodalton
kME	Module eigengene connectivity
LT	Low temperature (treatment)
ME	Module eigengene
MEME	Multiple EM for Motif Elicitation
ML	Maximum likelihood
mRNA	Messenger RNA
MUSCLE	Multiple Sequence Comparison by Log-Expectation
MYB	Myeloblastosis
NAC	NAM, ATAF1/2, and CUC2
NCBI	National Center for Biotechnology Information
NGDC	National Genomics Data Center
Os	*Oryza sativa*
PCF	PROLIFERATING CELL FACTOR
pI	Isoelectric point
PPI	Protein–protein interaction
qRT-PCR	Quantitative reverse-transcription PCR
RNA-seq	RNA sequencing
Sl	*Solanum lycopersicum*
SMART	Simple Modular Architecture Research Tool
SRA	Sequence Read Archive
St	*Solanum tuberosum*
*StSP6A*	*Solanum tuberosum* SELF-PRUNING 6A
STRING	Search Tool for the Retrieval of Interacting Genes/Proteins
TCP	TEOSINTE BRANCHED1/CYCLOIDEA/PROLIFERATING CELL FACTOR
TF	Transcription factor
UTR	Untranslated region
VST	Variance-stabilizing transformation
WGCNA	Weighted gene co-expression network analysis
Zm	*Zea mays*
