# Supplementary material for: Genome-Wide Identification of the TCP Gene Family in Tetraploid Potato and Analysis of Their Potential Roles in Tuber Development
Source: Int J Mol Sci. 2026 Oct 10;27(20):8995. doi: 10.3390/ijms27208995 (PMC13704912; doi:10.3390/ijms27208995)
Supplement: Supplementary file 1 [file ijms-27-08995-s001.zip › Figure S1 Sample clustering and developmental stages.pdf]

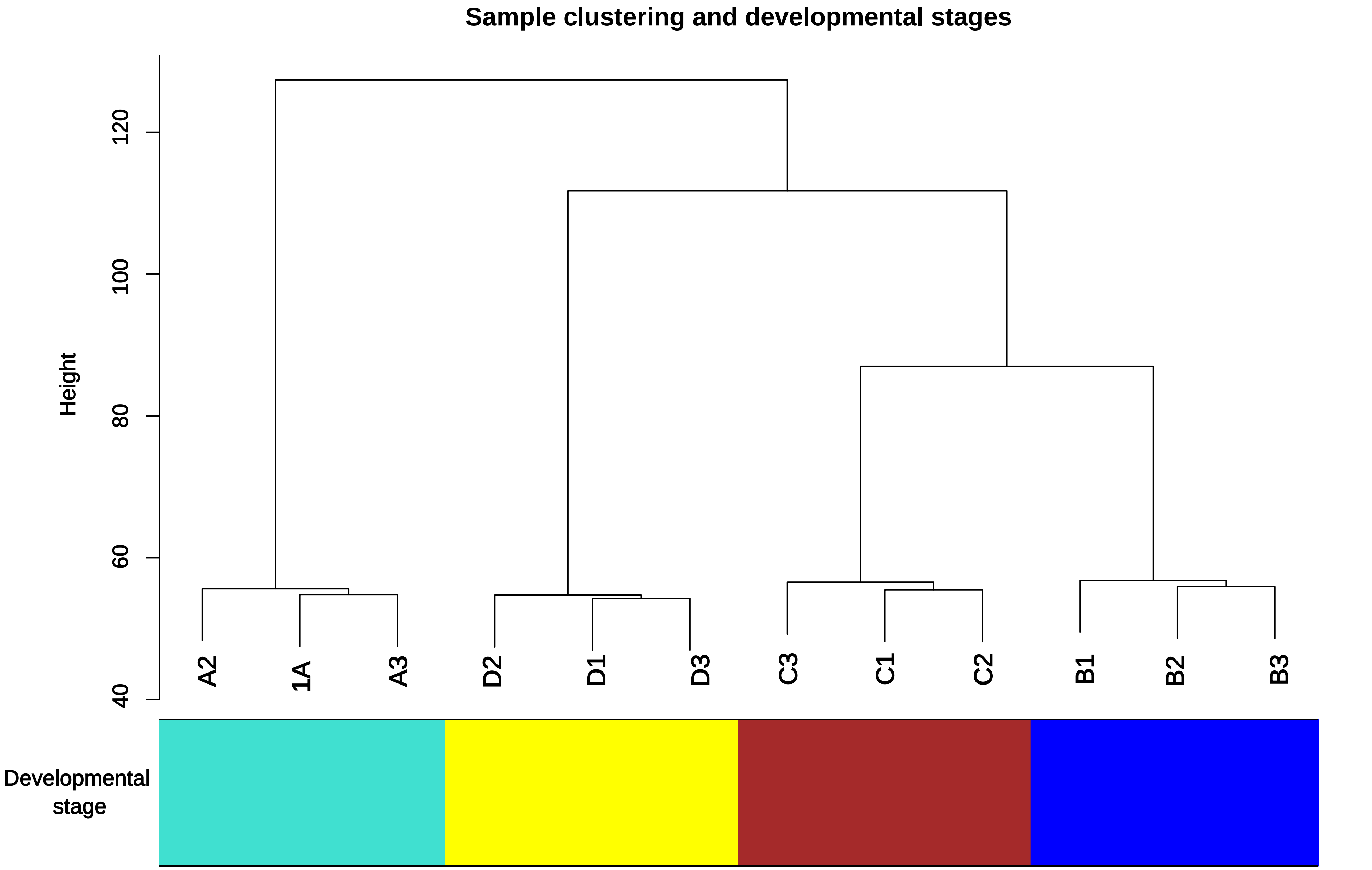

Sample clustering and developmental stages
Height
120
100
80
60
40
A2
1A
A3
D2
D1
D3
C3
C1
C2
B1
B2
B3
Developmental stage
