# Supplementary material for: Genome-Wide Identification of the TCP Gene Family in Tetraploid Potato and Analysis of Their Potential Roles in Tuber Development
Source: Int J Mol Sci. 2026 Oct 10;27(20):8995. doi: 10.3390/ijms27208995 (PMC13704912; doi:10.3390/ijms27208995)
Supplement: Supplementary file 1 [file ijms-27-08995-s001.zip › Figure S2 Selection of the soft-thresholding power.pdf]

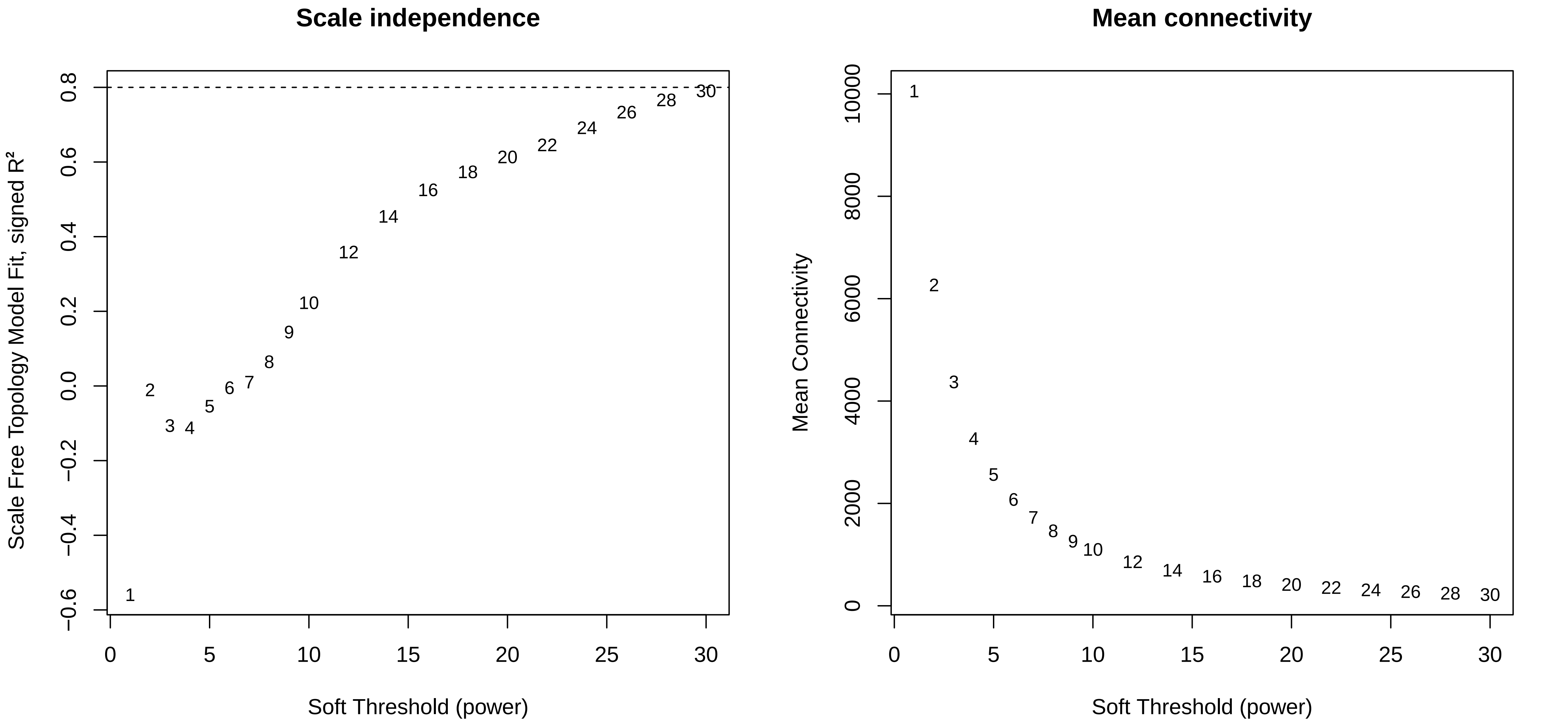
Scale independence
Scale Free Topology Model Fit, signed R²
Soft Threshold (power)
Mean connectivity
Mean Connectivity
Soft Threshold (power)
