# Supplementary material for: Genome-Wide Identification of the TCP Gene Family in Tetraploid Potato and Analysis of Their Potential Roles in Tuber Development
Source: Int J Mol Sci. 2026 Oct 10;27(20):8995. doi: 10.3390/ijms27208995 (PMC13704912; doi:10.3390/ijms27208995)
Supplement: Supplementary file 1 [file ijms-27-08995-s001.zip › Figure S3 Gene clustering dendrogram and module color assignment.pdf]

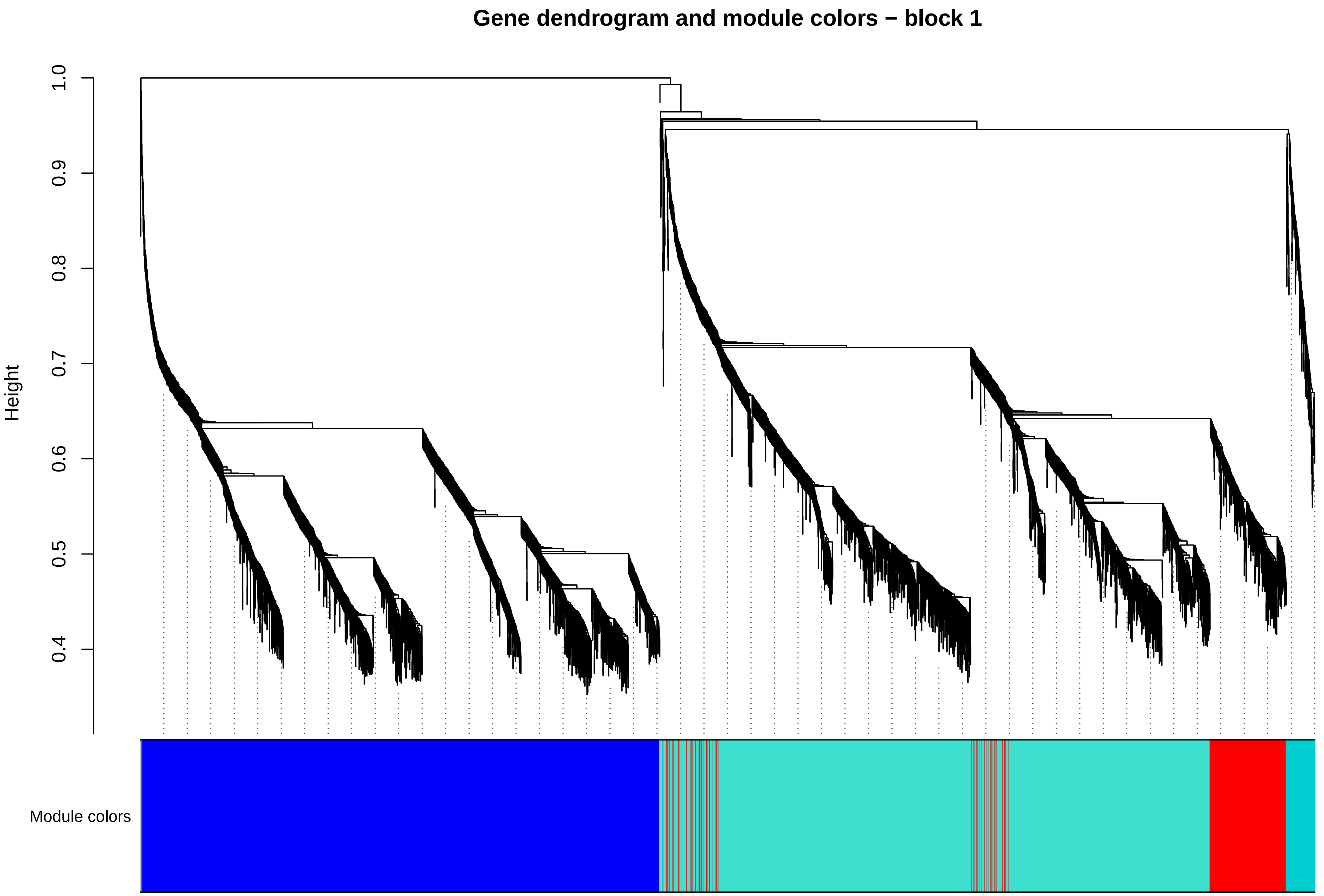

Gene dendrogram and module colors − block 1
Height
1.0
0.9
0.8
0.7
0.6
0.5
0.4
Module colors

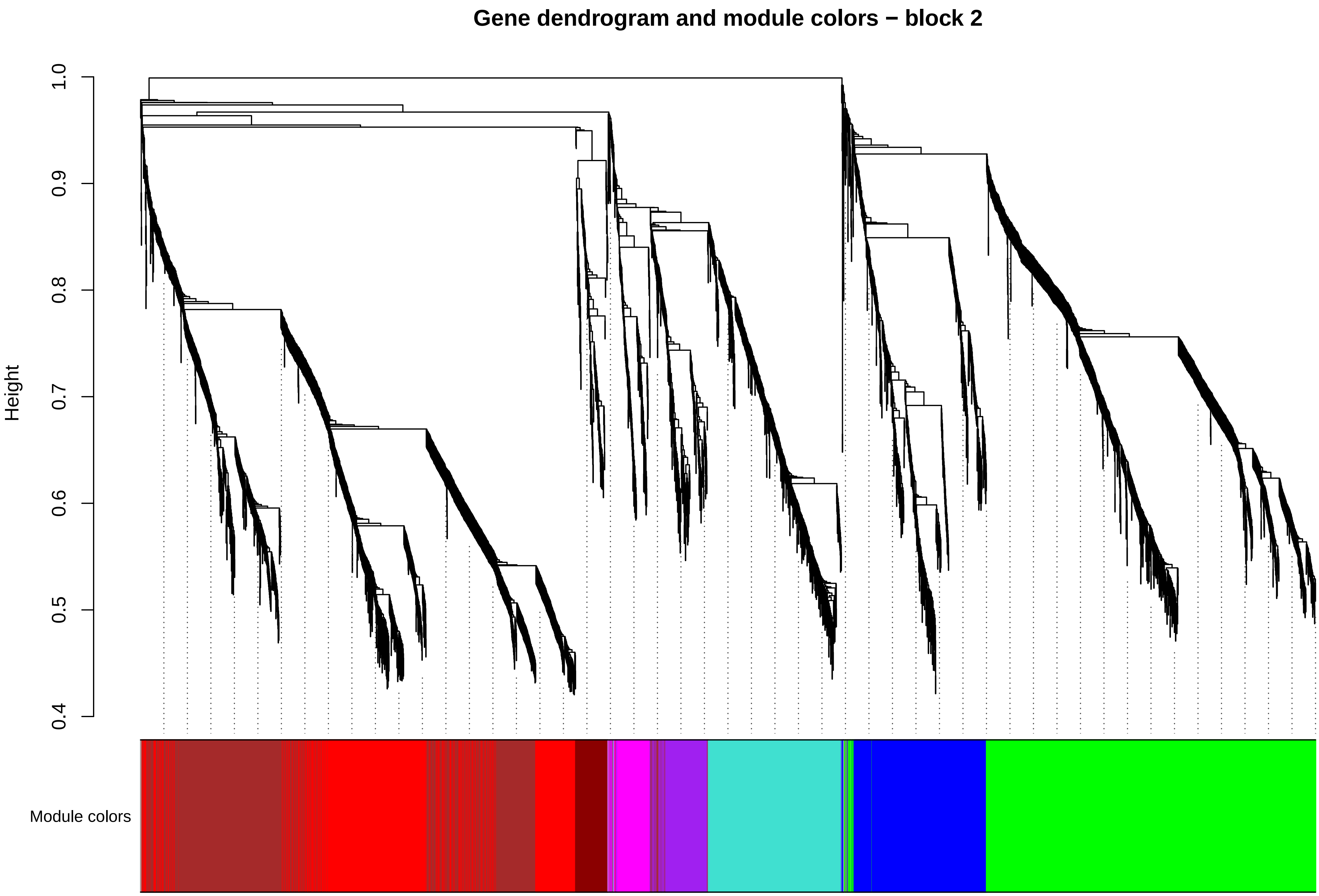
Gene dendrogram and module colors − block 2
Height
1.0
0.9
0.8
0.7
0.6
0.5
0.4
Module colors

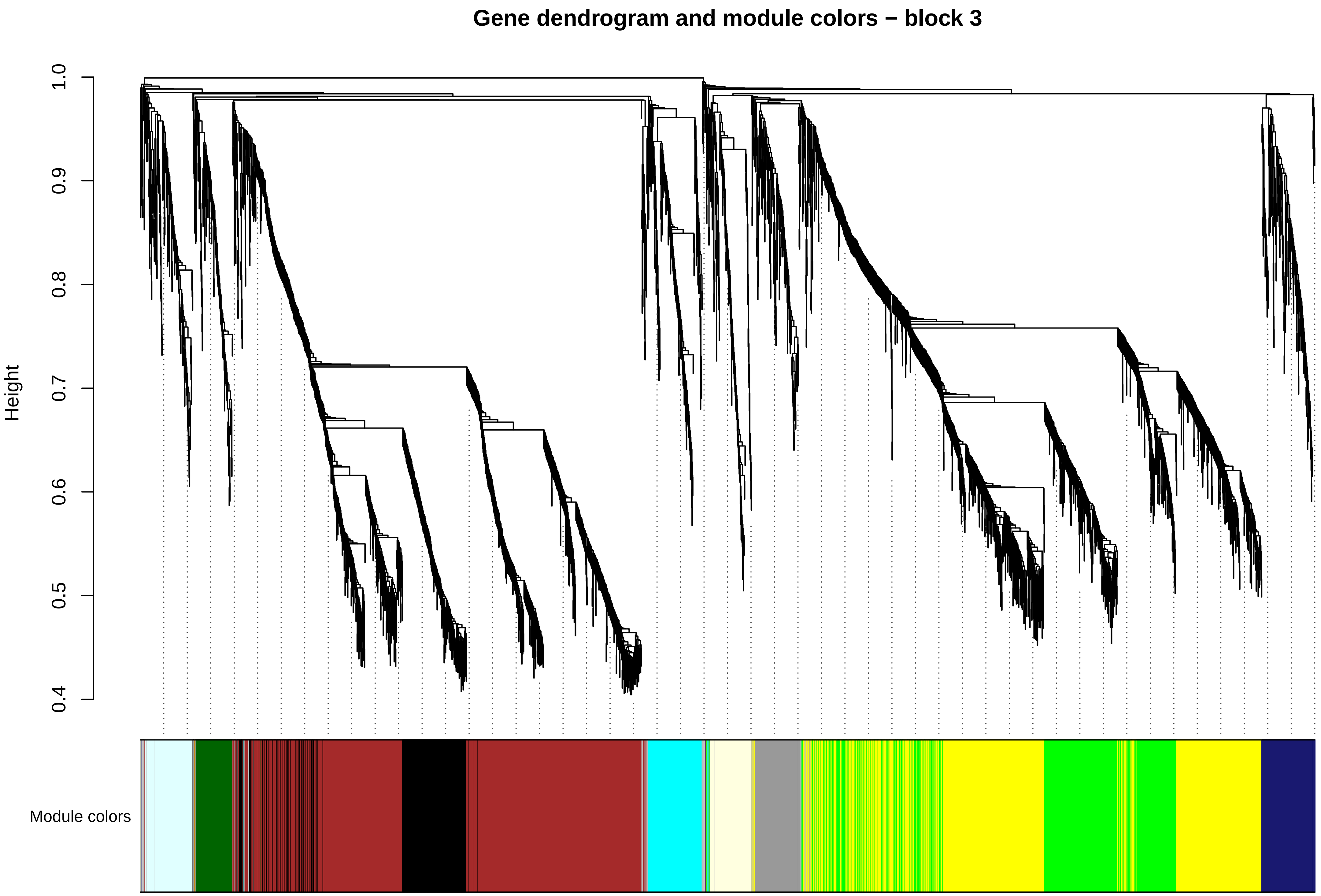

Gene dendrogram and module colors − block 3
Height
1.0
0.9
0.8
0.7
0.6
0.5
0.4
Module colors

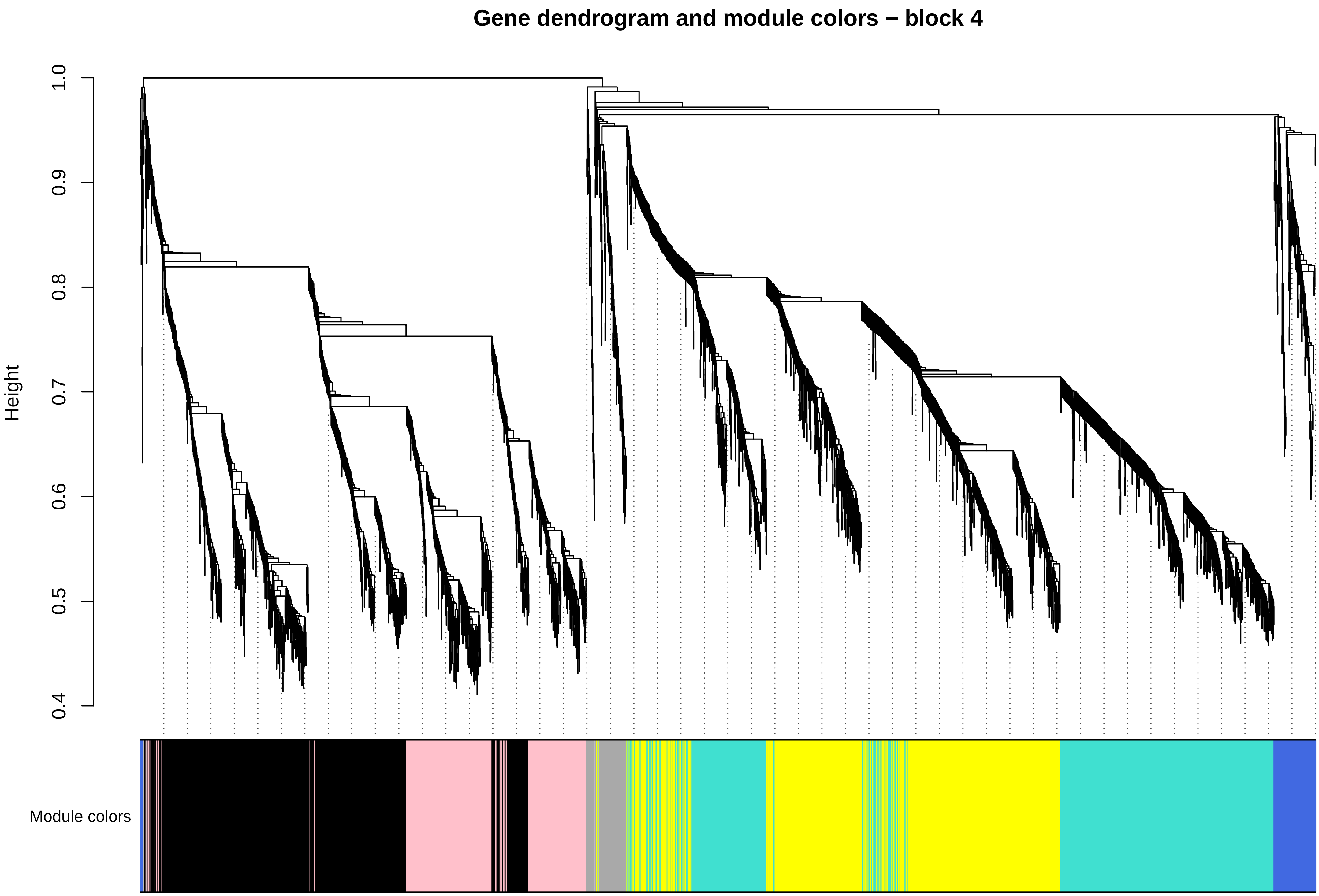
Gene dendrogram and module colors − block 4
Height
1.0
0.9
0.8
0.7
0.6
0.5
0.4
Module colors

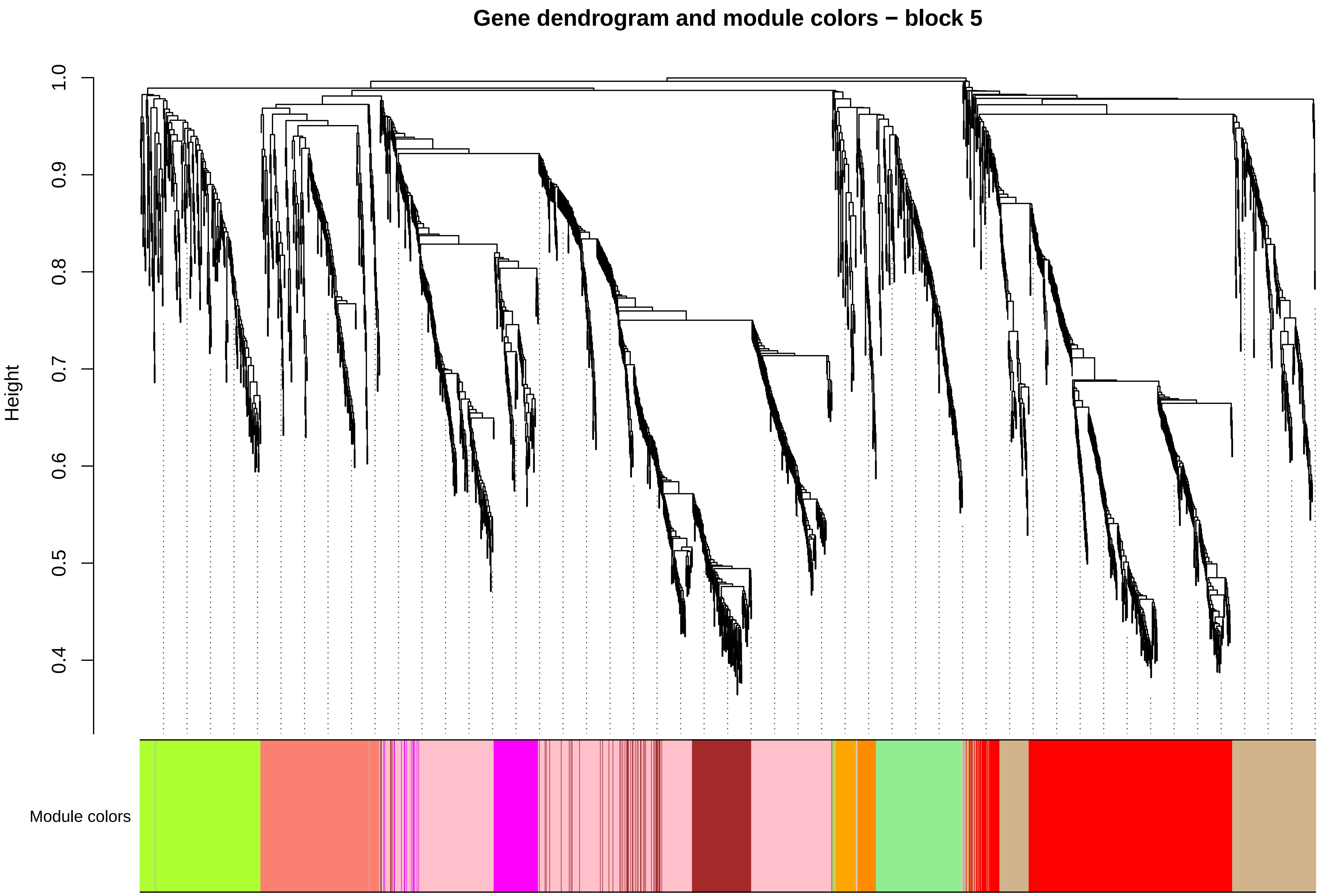

Gene dendrogram and module colors − block 5
Height
1.0
0.9
0.8
0.7
0.6
0.5
0.4
Module colors
