# Supplementary material for: Genome-Wide Identification of the TCP Gene Family in Tetraploid Potato and Analysis of Their Potential Roles in Tuber Development
Source: Int J Mol Sci. 2026 Oct 10;27(20):8995. doi: 10.3390/ijms27208995 (PMC13704912; doi:10.3390/ijms27208995)
Supplement: Supplementary file 1 [file ijms-27-08995-s001.zip › Supplementary Materials.pdf]

Supplementary Materials

Figure S1 Sample clustering and developmental stages

Figure S2 Selection of the soft-thresholding power

Figure S3 Gene clustering dendrogram and module color assignment

Table S1 Physicochemical characteristics and subcellular localization of StTCP proteins in tetraploid potato.

Table S2 TCP protein sequences of five species (potato, tomato, rice, maize, and Arabidopsis)

Table S3 Motifs sequence of the StTCP gene family in tetraploid potato.

Table S4 The 2-kb promoter sequences of the 57 StTCP genes are provided

Table S5 Statistics of the cis-acting elements in the StTCP gene promoters.

Table S6 Gene duplication of *StTCP* genes.

Table S7 Temporal expression profiles of genes in potato cultivar 'Qingshu 9' across different tissues (leaf and root) under high- and low-temperature treatments

Table S8 Expression levels of differentially expressed StTCP genes at different tuber developmental stages of potato.

Table S9 Gene Statistics for All Modules
